# On the importance of predictor choice, modelling technique, and number of pseudo‐absences for bioclimatic envelope model performance

**DOI:** 10.1002/ece3.6859

**Published:** 2020-10-16

**Authors:** Mirza Čengić, Jasmijn Rost, Daniela Remenska, Jan H. Janse, Mark A. J. Huijbregts, Aafke M. Schipper

**Affiliations:** ^1^ Department of Environmental Science Institute for Water and Wetland Research Radboud University Nijmegen The Netherlands; ^2^ PBL Netherlands Environmental Assessment Agency The Hague The Netherlands; ^3^ Netherlands eScience Center Amsterdam The Netherlands

**Keywords:** biomod2, climate change, ecological niche modeling, model performance, model transferability, species distribution modeling (SDM)

## Abstract

Bioclimatic envelope models are commonly used to assess the influence of climate change on species' distributions and biodiversity patterns. Understanding how methodological choices influence these models is critical for a comprehensive evaluation of the estimated impacts. Here we systematically assess the performance of bioclimatic envelope models in relation to the selection of predictors, modeling technique, and pseudo‐absences. We considered (a) five different predictor sets, (b) seven commonly used modeling techniques and an ensemble model, and (c) three sets of pseudo‐absences (1,000 pseudo‐absences, 10,000 pseudo‐absences, and the same as the number of presences). For each combination of predictor set, modeling technique, and pseudo‐absence set, we fitted bioclimatic envelope models for 300 species of mammals, amphibians, and freshwater fish, and evaluated the predictive performance of the models using the true skill statistic (TSS), based on a spatially independent test set as well as cross‐validation. On average across the species, model performance was mostly influenced by the choice of predictor set, followed by the choice of modeling technique. The number of the pseudo‐absences did not have a strong effect on the model performance. Based on spatially independent testing, ensemble models based on species‐specific nonredundant predictor sets revealed the highest predictive performance. In contrast, the Random Forest technique yielded the highest model performance in cross‐validation but had the largest decrease in model performance when transferred to a different spatial context, thus highlighting the need for spatially independent model evaluation. We recommend building bioclimatic envelope models according to an ensemble modeling approach based on a nonredundant set of bioclimatic predictors, preferably selected for each modeled species.

## Graphical abstract

Bioclimatic envelope models are commonly used to assess the influence of climate change on species' distributions and biodiversity patterns. We systematically evaluated the performance of bioclimatic envelope models in relation to the selection of predictors, modelling technique, and pseudo‐absences. Model performance was mostly influenced by the choice of predictor set, followed by the choice of modelling technique, and based on our results, we recommend building bioclimatic envelope models according to an ensemble modelling approach based on a nonredundant set of bioclimatic predictors, preferably selected for each modelled species.

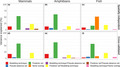

## 1 INTRODUCTION

In the face of ongoing climate change it is crucial to quantify and understand its impacts on biodiversity. Threats of future climate change to biodiversity are commonly quantified with bioclimatic envelope models, that is, species distribution models (SDMs) that link species presence records with climatic variables to project the future distribution of species in response to climate change (Elith & Leathwick, 2009; Guisan & Thuiller, 2005; Pacifici et al., 2015; Pearson & Dawson, 2003; Thomas et al., 2004). Methodological choices made during the development of SDMs vary widely and may have considerable influence on the model output (Araújo et al., 2005, 2019; Araújo & Peterson, 2012; Barry & Elith, 2006; Pearson et al., 2006; Thuiller et al., 2019). This points at a clear need to understand how methodological choices affect the performance of SDMs in general and bioclimatic envelope models in particular.

Key methodological aspects that influence SDMs include the selection of predictor variables, selection of modeling technique, and selection of pseudo‐absence data (Araújo et al., 2019; Brun et al., 2019; Merow et al., 2014; Thuiller et al., 2019; Warren et al., 2019). Although there is consensus that predictor variables should be ecologically relevant to the species of concern, often this is challenging due to data limitations or because the ecological requirements of the species are not sufficiently known (Araújo et al., 2019). Many studies therefore rely on a (semi‐)automated selection of complementary (i.e., not too highly correlated) predictors from a broader set of variables that are expected to be relevant (Barbet‐Massin & Jetz, 2014; Bradie & Leung, 2017; Dormann et al., 2013; Petitpierre et al., 2017).

When it comes to the choice of modeling technique, there is a lack of consensus as to which technique is most suited for which SDM purpose (Araújo et al., 2019; Benito et al., 2013). Some studies build models based on machine‐learning approaches such as Random Forest or MaxEnt (Bahn & McGill, 2013; Elith et al., 2011; Guisan et al., 2007), while others apply regression‐based approaches (Guisan et al., 2002; Guisan & Zimmermann, 2000; Li & Wang, 2013) or prefer an ensemble of several modeling techniques (Araújo & New, 2007; Marmion et al., 2009; Rapacciuolo et al., 2012). There is evidence, however, that ensemble modeling is the preferred choice if the SDMs are to be used for projections, which are key in future climate threat assessments (Araújo et al., 2005).

The selection of pseudo‐absence or background data is a third key consideration in SDM building, given that the majority of species observations concern presence‐only data (Ponder et al., 2001). Relevant aspects include the number of pseudo‐absences as well as their spatial distribution (i.e., extent and geographic stratification), which may affect model performance as well as the relative importance of predictor variables (Barbet‐Massin et al., 2012; Phillips et al., 2009; VanDerWal et al., 2009).

The impact of predictor set, modeling technique, and pseudo‐absence selection on the performance of bioclimatic envelope models has been studied in isolation (Barbet‐Massin et al., 2012; Beaumont et al., 2005; Moreno‐Amat et al., 2015; Pearson et al., 2006; Pliscoff et al., 2014) or for two of the three factors (Brun et al., 2019; Bucklin et al., 2015; Dormann et al., 2008; Jarnevich et al., 2017; Petitpierre et al., 2017; Verbruggen et al., 2013; Warren et al., 2019). However, to our knowledge, no study so far has systematically evaluated the combined effect of predictor set, modeling technique, and pseudo‐absence selection on the performance of bioclimatic envelope models. Here we seek to fill that gap by systematically varying the choice of predictor set, modeling technique and number of pseudo‐absences and evaluating the performance of the resulting bioclimatic envelope models.

We evaluated five sets of bioclimatic predictors, seven modeling techniques plus a consensus ensemble model, and three sets of pseudo‐absences. We selected a sample of 100 species from each of three major taxonomic groups, that is, mammals, amphibians, and freshwater fish, as the performance of bioclimatic envelope models has proven to differ among species groups (Heikkinen et al., 2012). For each species, we fitted a bioclimatic envelope model for each combination of predictor set, modeling technique, and pseudo‐absence set. We evaluated the models with cross‐validation (random split sample) and based on their transferability to a spatially independent region, made up by different biomes (for mammals and amphibians) or different catchments (for fish). Thus, we test both model accuracy (the ability of the model to predict well within the space and time covered by the input data) and model generality (the ability of the model to predict well in another region or time), whereby the latter is more critical for bioclimatic envelope models given their aim to forecast changes (Araújo et al., 2019).

We asked the following research questions:

1. How sensitive is the performance of bioclimatic envelope models to the choice of predictor set, modeling technique, and number of pseudo‐absences?
2. Which combination of predictor set, modeling technique, and pseudo‐absence set yields the highest model performance?
3. To what extent are the outcomes contingent on the evaluation method, that is, independent testing as opposed to cross‐validation?

## 2 MATERIALS AND METHODS

### 2.1 Species data

We retrieved species presence data from expert range maps provided by the IUCN (IUCN, 2018), similar to other recent studies that developed and applied bioclimatic envelope models (Hof et al., 2018; Visconti et al., 2016). The IUCN Red List assesses the conservation status of individual (sub)species and provides range maps that characterize the global distribution of species of several taxonomic groups. We preferred using range maps over global point records databases (e.g., GBIF), as range maps are expected to be less spatially biased (Fourcade, 2016; Merow et al., 2016). We included not only areas where the species are extant but also areas where the species are currently extinct, assuming that extinction was driven by factors other than climate change (Faurby & Svenning, 2015). We obtained data for each species by rasterizing its IUCN range map to a resolution of 5 arc‐minutes and taking each raster cell that falls inside of the range of the species as a presence record.

We retrieved pseudo‐absences from an area outside the range maps, but potentially within reach of the species (Elith & Leathwick, 2009; Phillips et al., 2009). For mammals and amphibians, we used the terrestrial biomes encompassing the species' ranges to sample pseudo‐absences (Vences & Köhler, 2008). For freshwater fish, we used the catchments encompassing the ranges (Berra, 2001). The terrestrial biomes map was derived from the Terrestrial Ecoregions of the World map (Olson et al., 2001). The catchments map was constructed by combining the hydrography of Hydrosheds and Hydro1k at 30 arc‐seconds and derived using the “Basin” function in ArcGIS, with a total of 152,739 catchments (Barbarossa et al., 2018). We rasterized the biome and catchment maps at a 5 arc‐minutes resolution to match the spatial resolution of the rasterized species ranges. For each species, we then randomly selected an initial set of maximum 100,000 pseudo‐absence raster cells outside the range of that species but within the surrounding biomes or catchments.

Next, we split the species data into training and test data. We created two test datasets for each species, that is, a cross‐validation set and a spatially independent set. For the cross‐validation set, we randomly took 80% of the data for model training and used the remaining 20% for model testing, without considering the spatial structure of the data. To create the spatially independent test set, we sorted the biomes (for mammals and amphibians) or catchments (for fish) based on the number of presence and pseudo‐absence points in the area. The records in the biomes or catchments were then alternately assigned to the training or test set, starting with the training set to ensure that the training set would have the largest number of records, resulting in at least half of the species data assigned to the training data set. We then selected species for which both the training and test set included at least 100 points in the species range and 10,000 pseudo‐absences. From this list of species, we randomly selected 100 species from each taxonomic group for our analysis. A list of species is provided in Table S1.

### 2.2 Predictors, modeling techniques, and pseudo‐absence sets

#### 2.2.1 Predictors

We retrieved the climate data for fitting the bioclimatic envelope models from the global 5 arc‐minutes resolution Worldclim 1.4 set (Hijmans et al., 2005). The Worldclim dataset contains 19 bioclimatic variables that represent aspects of temperature and precipitation that are considered particularly relevant for species and ecosystems. From this dataset we compiled five different sets of predictors. The larger the set of predictor variables, the more likely that it includes ecologically meaningful variables for the species of concern, yet at the expense of an increased risk of model overfitting and spurious relationships (Barbet‐Massin & Jetz, 2014; Merow et al., 2014). We defined four generic predictor sets consisting of an increasing number of variables and a fifth species‐specific set of nonredundant variables specific to each species, as follows:

1. A predictor set consisting of two variables representing mean climate, that is, annual mean temperature and annual precipitation (bio1 and bio12).
2. A predictor set consisting of four variables, representing mean climate and seasonality, that is, annual mean temperature and annual precipitation and the seasonality of temperature and precipitation (bio1, bio4, bio12 and bio15).
3. A set of nonredundant bioclimatic variables identified based on multicollinearity within the entire dataset (i.e., global extent). To that end, we calculated variance inflation factors (VIFs) using the “vifstep” function in R package “usdm” (Naimi et al., 2014). We excluded a variable if its VIF value was above 10, starting with the variable with the highest value (Duque‐Lazo et al., 2016; Zuur et al., 2010). This resulted in the following 10 remaining predictors: bio2, bio3, bio4, bio8, bio9, bio13, bio14, bio15, bio18 and bio19.
4. A predictor set consisting of all 19 bioclimatic predictors, reflecting the strategy of including a broad set of possible bioclimatic predictor variables and relying on the modeling technique to identify relevant variables or penalize models with collinear ones (Brun et al., 2019; Dormann et al., 2013).
5. A species‐specific set of nonredundant variables, identified by excluding collinear variables based on a variance inflation factor (VIF) larger than 10 calculated based on the input data specific to each individual species. We calculated the VIF values according to the approach as explained for predictor set 3. The number of remaining variables for each species is given in Table S1.

#### 2.2.2 Modeling techniques

We used seven modeling techniques to fit the bioclimatic models, including one classification method (Classification Tree Analysis, CTA), three regression techniques (Generalized Linear Model, GLM; Generalized Additive Model, GAM; Multivariate Adaptive Regression Splines, MARS) and three machine‐learning techniques (Generalized Boosted Model, GBM; Random Forest, RF; Maximum Entropy, MaxEnt). These techniques are commonly used in bioclimatic envelope modelling (Araújo et al., 2005; Bahn & McGill, 2013; Barbet‐Massin & Jetz, 2014; Beaumont et al., 2016; Jeschke & Strayer, 2008). Although various studies already indicated that some of these techniques perform better than others (Araújo et al., 2005; Bahn & McGill, 2013; Benito et al., 2013; Guisan et al., 2007; Heikkinen et al., 2012; Rapacciuolo et al., 2012; Ruiz‐Navarro et al., 2016), this was typically tested in cross‐validation, and their transferability in space has not yet been extensively tested. Besides using the individual techniques, we evaluated also an ensemble model consisting of the average of the fitted models across the seven techniques (Araújo & New, 2007). We selected individual models only if their true skill statistic (TSS) value was higher than 0.7, and we weighted the models according to their TSS value (Marmion et al., 2009).

#### 2.2.3 Pseudo‐absences

We selected three sets of pseudo‐absences from the full set of pseudo‐absences available in the training data set. The first pseudo‐absence set consisted of 10,000 randomly selected pseudo‐absences. For the second set, 1,000 pseudo‐absences were randomly selected. For the last set, the number of selected pseudo‐absences was equal to the number of presences available for model fitting (i.e., in the training dataset). These sets were selected based on recommendations regarding the number of pseudo‐absences to be used for different modeling techniques (Barbet‐Massin et al., 2012). We applied equal weighting of presences and pseudo‐absences in model fitting, as recommended by Barbet‐Massin et al. (2012).

#### 2.2.4 Model fitting and evaluation

We fitted the models using the “biomod2” package in R (Thuiller et al., 2016), which comprises the most commonly used techniques in species distribution modeling. We kept the default values of tuning and fitting parameters for each modeling technique, as given in the biomod2 package. The combination of five predictor sets, seven modeling techniques and one ensemble, and three pseudo‐absence sets resulted in 120 fitted models for each species, hence a total of 36,000 models for all 300 species. We evaluated the bioclimatic envelope models based on their transferability to the spatially independent test set. For comparison, we also performed cross‐validation within the training dataset that includes all biomes and catchments. We quantified model performance based on the TSS value (Allouche et al., 2006).

To assess the sensitivity of model performance to the number of predictors, modeling technique and pseudo‐absences selection, we fitted linear mixed effects models relating TSS values to these modeling choices using the “lmer” function from the “lme4” package in R (Bates et al., 2015). We fitted a separate model per taxonomic group and per evaluation method (spatially independent testing or cross‐validation) relating the TSS values to predictor set (factor with five levels), modeling technique (factor with eight levels), and pseudo‐absences set (factor with three levels), including their interactions. We included species as a random effect (intercept) in order to account for having repeated measures within species (i.e., the 120 combinations of predictor set, modeling techniques and pseudo‐absences set). Because model performance and transferability may depend on the degree of similarity in environmental conditions between training and extrapolation (Qiao et al., 2019), we added a measure of environmental niche overlap between the training and testing data as an additional fixed effect. To that end, we calculated the overlap in the environmental space between the training and testing datasets based on the predictor set with four bioclimatic variables, measured by the Jaccard similarity index (Jost, 2006) with the R package hypervolume (Blonder et al., 2014). As the overlap estimate can be inflated with the addition of more dimension (Blonder et al., 2014), we used the overlap values derived from the predictor set containing four variables with normalized values. Per taxonomic group and validation method (spatially independent versus cross‐validation), we selected the best model based on the lowest Akaike information criterion (AIC). To quantify the contribution of each random and fixed effect to the variability in model performance, we retrieved the percentage of explained variance using the “r2beta” function with the standardized generalized variance approach from the “r2glmm” package (Jaeger et al., 2017).

## 3 RESULTS

### 3.1 Variation in model performance

**Figure 1:**
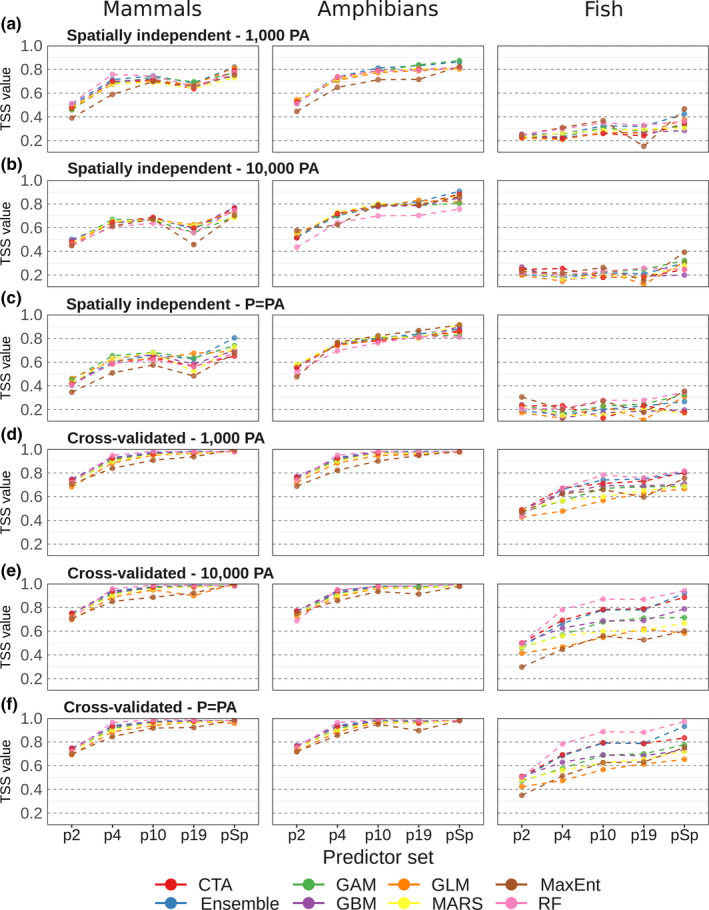
Mean TSS values for each combination of predictor set and modeling technique for spatially independent testing (panels a–c) and cross‐validation (d–f). The results are given for all three pseudo‐absence (PA) datasets, including 1,000 PA (panels a and d), 10,000 PA (panels b and e), and a number equal to the number presences (P = PA, panels c and f). Predictor sets include two variables (p2), four variables (p4), a nonredundant set of 10 variables (p10), all 19 bioclimatic variables (p19), and species‐specific nonredundant sets (pSp). CTA = Classification Tree Analysis; GAM = Generalized Additive Model; GBM = Generalized Boosted Model; GLM = Generalized Linear Model; MARS = Multivariate Adaptive Regression Splines; MaxEnt = Maximum Entropy; RF = Random Forest. Mean TSS values and corresponding 5 and 95 percentiles are given in Tables S1 and S3

**Figure 2:**
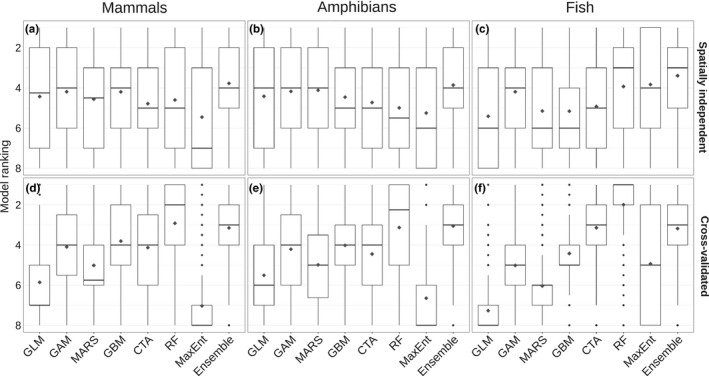
Modeling technique ranking based on TSS values, shown as distribution of ranks across the species, pseudo‐absence sets and predictor sets. Modeling technique rankings are given for Mammals (panels a and d), Amphibians (panels a and e), and Fish (panels c and f), both in spatially independent testing (panels a‐c) and cross‐validation (panels d‐f). Model ranking of 1 is the highest ranked model (highest TSS value), while 8 is the lowest ranked model. Boxplots show median rank values, quartiles, and 1.5 times of the interquartile distance of the ranks; diamonds represent means. CTA = Classification Tree Analysis; GAM = Generalized Additive Model; GBM = Generalized Boosted Model; GLM = Generalized Linear Model; MARS = Multivariate Adaptive Regression Splines; MaxEnt = Maximum Entropy; RF = Random Forest

TSS values derived with spatially independent data ranged from 0.35 to 0.81 for mammals, from 0.44 to 0.92 for amphibians, and from 0.11 to 0.47 for fish (Figure 1). TSS values obtained in spatially independent testing were consistently lower than those obtained in cross‐validation, which ranged from 0.68 to 1 for mammals, from 0.69 to 1 for amphibians, and from 0.29 to 0.97 for fish. We found the lowest average TSS values for fish, while amphibians and mammals had higher values. In general, a species‐specific set of nonredundant variables resulted in the highest model performance. In addition, model performance generally increased with the number of predictors, whereby the largest increase in model performance occurred between the sets with two and four predictors. Models based on the full set of 19 bioclimatic predictors performed no or only marginally better than models based on a nonredundant set of 10 predictors. On average, the ensemble model performed best in the spatially independent testing, while in the cross‐validation the Random Forest technique resulted in the highest predictive power (Figures 1 and 2). The MaxEnt models had the lowest performance on average both in the spatially independent testing and the cross‐validation (Figure 2), which was most evident for the spatially independent evaluation of the mammal species models (Figure 1). There were no evident differences in model performance between the three pseudo‐absence sets (Figures S1 and S2).

### 3.2 Sensitivity to predictor, modeling technique, and pseudo‐absences selection

**Figure 3:**
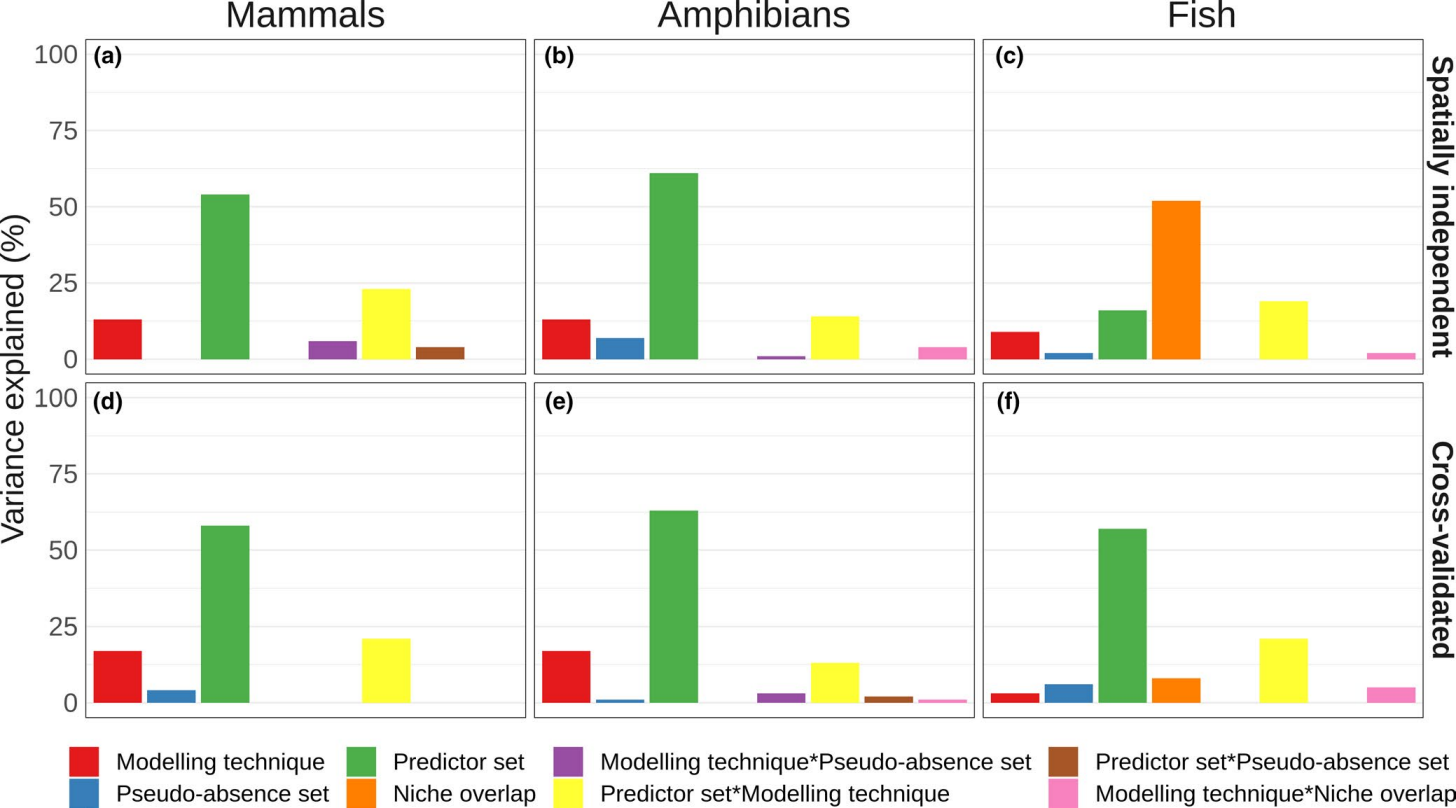
Proportions of variance in model performance explained by predictor set, modeling technique, number of pseudo‐absences and niche overlap. Proportions of explained variance are given for Mammals (panels a and d), Amphibians (panels b and e), and Fish (panels c and f), both in spatially independent testing (panels a–c) and cross‐validation (panels d–f). Proportions of variance explained were rescaled to sum to 100% across the factors. Underlying data is in Table S4

Model performance was mostly related to the choice of predictors set, both for spatially independent validation and cross‐validation (Figure 3). The only exception was the performance of the fish models in spatially independent validation, which was mostly related to the overlap between the training and testing data (niche overlap). The choice of modeling technique was the second most important factor explaining variation in model performance, in interaction with the predictor set, or on its own. The number of pseudo‐absences explained only a very small part of the variation in model performance. This ranking did not change when we excluded the smallest and largest predictor sets (Figure S3).

## 4 DISCUSSION

In this study, we evaluated the sensitivity of bioclimatic envelope model performance to choices in predictor set, modeling technique, and number of pseudo‐absences. Based on spatially independent testing, that is, the preferred testing strategy for bioclimatic envelope models (Araújo et al., 2005; Bahn & McGill, 2013; Roberts et al., 2017), we found that the highest model performance is obtained if the selection of bioclimatic predictors is tailored to the species of concern (Figure 1). In addition, we found that model performance generally improves by increasing the number of predictors (Figure 1). In part, this may reflect a mere effect of chance, as each predictor (even a random one) may explain a bit of additional variation just by chance (Fourcade et al., 2018). However, we also found that the relative increase in model performance became smaller or even declined slightly when the full set with 19 predictors was used rather than more parsimonious predictor sets with 10 variables. Our findings thus indicate that the set of 19 predictors can be profitably reduced to a (preferably species‐specific) nonredundant set based on an evaluation of multicollinearity. This would reduce the risk of erroneous model inference or projections arising from collinearity issues, due to the inflated regression parameters or models being projected to variables with different levels of collinearity (Dormann et al., 2013).

Our results further indicate that an ensemble model is the preferred option for bioclimatic envelope modeling, as the ensemble generally performed best based on the spatially independent testing. Although individual modeling techniques may outperform the ensemble model in some cases, overall the ensemble model demonstrated a higher and a more consistent performance compared to the individual techniques (Figure 2). This is in line with other studies that demonstrated that model ensembles may outperform or give more robust estimates compared to the individual modeling techniques that are used to build a consensus model (Araújo & New, 2007; Buisson et al., 2010; Crimmins et al., 2013; Marmion et al., 2009).

The Random Forest models generally performed best in cross‐validation but showed a clear drop in performance when evaluated based on spatially independent data, both in absolute terms and compared to other modeling techniques (Figure 2, Table S2). High performance of RF and other more complex modeling techniques in cross‐validation is commonly observed, as more complex techniques tend to fit the relationship closely to the data (Merow et al., 2014; Randin et al., 2006), but perform poorly when models are transferred into novel spatial or temporal contexts. Similar results for RF were shown in a study that compared random data partitioning with using temporally independent data for model evaluation, where RF performed well when assessed on internal cross‐validation, yet poorly when assessed with an independent dataset (Crimmins et al., 2013).

We found that MaxEnt models often performed relatively poorly (Figures 1 and 2), despite the fact that MaxEnt is a widely used modeling technique for SDMs (Warren & Seifert, 2011). Applying background data (i.e., sampled from the entire study area) rather than pseudo‐absences (i.e., sampled outside of the species' presence ranges), as recommended for MaxEnt (Phillips et al., 2006), did not result in an improvement in the performance of the MaxEnt models (Figure S4). Our findings are in line with previous studies indicating that MaxEnt is prone to overfitting and does not perform well when a model is transferred to a different spatial or temporal context (Peterson et al., 2007; Radosavljevic & Anderson, 2014). However, the performance of the MaxEnt models clearly increased when fitted based on the species‐specific input datasets (Figure 1), underlining the need to tune MaxEnt models to the species of concern (Elith et al., 2011; Radosavljevic & Anderson, 2014).

We found that the number of pseudo‐absences did not have a strong effect on model performance, in particular when compared with the effect of the predictor set. Although it has been recommended to tailor the number of pseudo‐absences to the technique of choice (Barbet‐Massin et al., 2012), our results indicate only a weak interactive effect of the number of pseudo‐absences and the modeling technique for mammals (Figure 3). Only for the amphibian models, there was a small effect of the number of pseudo‐absence on its own. Our results thus suggest that selecting a fixed number of pseudo‐absences for all species is a defensible choice (Bucklin et al., 2015; Petitpierre et al., 2017; Richmond et al., 2010).

Differences in model performance among the taxonomic groups could result from differences in the background regions from which the pseudo‐absences were selected. For mammals and amphibians the background region consisted of biomes encompassing the species' range, whereas for fish the background region was restricted to the catchments in which the species occurs. The smaller spatial extent of catchments and resulting smaller background area from which values are sampled might explain the lower mean TSS for fish, as a more restricted background region (i.e., smaller environmental gradients) typically reduces model performance (Leroy et al., 2018; Lobo et al., 2008; VanDerWal et al., 2009). The smaller background region for fish species may also explain the larger importance of environmental niche overlap between training and testing data in explaining the variation in TSS (Figure 3). Smaller spatial units to delineate spatially independent training and testing datasets may result in a larger variation in niche overlap between different species (Figure S5) hence a larger influence on variation in model performance.

The relatively low model performance for freshwater fish may also reflect that we fitted the models based on bioclimatic predictors representing air temperature and precipitation, whereas the distributions of freshwater fish is also influenced by other factors (Barbarossa et al., 2020; Knouft & Ficklin, 2017; McGarvey et al., 2018). However, two key factors underlying the occurrence of freshwater fish (i.e., water temperature and streamflow) depend on air temperature and precipitation (Barbarossa et al., 2018; Knouft & Ficklin, 2017), and it has indeed been demonstrated that bioclimatic variables can be effectively used as proxies for macro‐scale modeling of the distribution of freshwater species (Domisch et al., 2015; Frederico et al., 2014; McGarvey et al., 2018).

In conclusion, our study shows a clear impact of predictor and modeling technique selection on the performance of bioclimatic envelope models. Our findings indicate that bioclimatic envelope models are preferably built based on a species‐specific, nonredundant set of predictor variables, and an ensemble modeling approach. A pragmatic choice can be made for the number of pseudo‐absences, for example, based on runtime, as the number of pseudo‐absences appeared to be less influential on model performance. In contrast to the results of the spatially independent testing, cross‐validation pointed toward Random Forest as the preferred modeling technique. This finding highlights that cross‐validation does not necessarily identify the best combination of modeling technique and predictor set to establish a predictive model, as displayed by a drop in performance when comparing cross‐validation and spatially independent validation.

## CONFLICT OF INTERESTS

None declared.

## AUTHOR CONTRIBUTIONS

**Mirza Čengić:** Data curation (lead); formal analysis (lead); investigation (lead); methodology (lead); software (lead); validation (lead); visualization (lead); writing–original draft (equal); writing–review and editing (equal). **Jasmijn Rost:** Conceptualization (equal); formal analysis (equal); investigation (equal); methodology (equal); software (equal); visualization (equal); writing–original draft (lead). **Daniela Remenska:** Data curation (equal); software (equal). **Jan H. Janse:** Writing–review and editing (equal). **Mark A. J. Huijbregts:** Conceptualization (equal); methodology (equal); supervision (equal); writing–review and editing (equal). **Aafke M. Schipper:** Conceptualization (lead); methodology (equal); supervision (lead); writing–review and editing (lead).

## Supporting information

Appendix S1

## Data Availability

The species data used in this study are publicly available on the IUCN Red List website (https://www.iucnredlist.org/resources/spatial‐data‐download). Climate data retrieved from the WorldClim v1.4 database are publicly available on the WorldClim website (https://worldclim.org/data/v1.4/worldclim14.html). The code of the analysis is archived at https://doi.org/10.5281/zenodo.4022773 as the GitHub repository. With this code and the input data specified above, results in this paper can be reproduced.
