# Supplementary material for: On the importance of predictor choice, modelling technique, and number of pseudo‐absences for bioclimatic envelope model performance
Source: Ecol Evol. 2020 Oct 16;10(21):12307–17. doi: 10.1002/ece3.6859 (PMC7663074; doi:10.1002/ece3.6859)
Supplement: Supplementary file 1 — Appendix S1 [file ECE3-10-12307-s001.docx]

**Supplementary information**

**On the importance of predictor choice, modelling technique and number of pseudo-absences for bioclimatic envelope model performance**

Authors: Mirza Čengić^1‡*^, Jasmijn Rost^2‡^, Daniela Remenska^3^, Jan H. Janse^2^, Mark A.J. Huijbregts^1^, Aafke M. Schipper^1,2^

^1^ Department of Environmental Science, Institute for Water and Wetland Research, Radboud University Nijmegen, P.O. Box 9010, 6500 GL Nijmegen, The Netherlands

^2^ PBL Netherlands Environmental Assessment Agency, P.O. Box 30314, 2500 GH The Hague, The Netherlands

^3^ Netherlands eScience Center, Science Park 140, 1098 XG Amsterdam, The Netherlands

^‡^ Both authors contributed equally to this manuscript

^*^ Corresponding author: Mirza Čengić: m.cengic@science.ru.nl

**Contents**

Figure S1: Mean TSS and standard error of each combination of predictor and pseudo-absence set per modelling technique in spatially independent testing.

Figure S2: Mean TSS and standard error of each combination of predictor and pseudo-absence set per modelling technique in cross-validation.

Figure S3: Proportions of variance explained in model performance, excluding the predictor sets with 2 and 19 variables.

Figure S4: MaxEnt model performance comparison based on pseudo-absences and background data.

Figure S5: Distribution of environmental overlap values between the training and test datasets.

Table S1: List of 100 species per group that were considered for modelling, with the number of variables that remained after stepwise elimination of variables with VIF threshold of 10.

Table S2: Model performance measured by mean TSS value in spatially independent assessment for each combination of modelling technique, predictor set and pseudo-absences selection.

Table S3: Model performance measured by mean TSS value in cross-validation for each combination of modelling technique, predictor set and pseudo-absences selection.

Table S4: Proportions of variance explained in model performance.


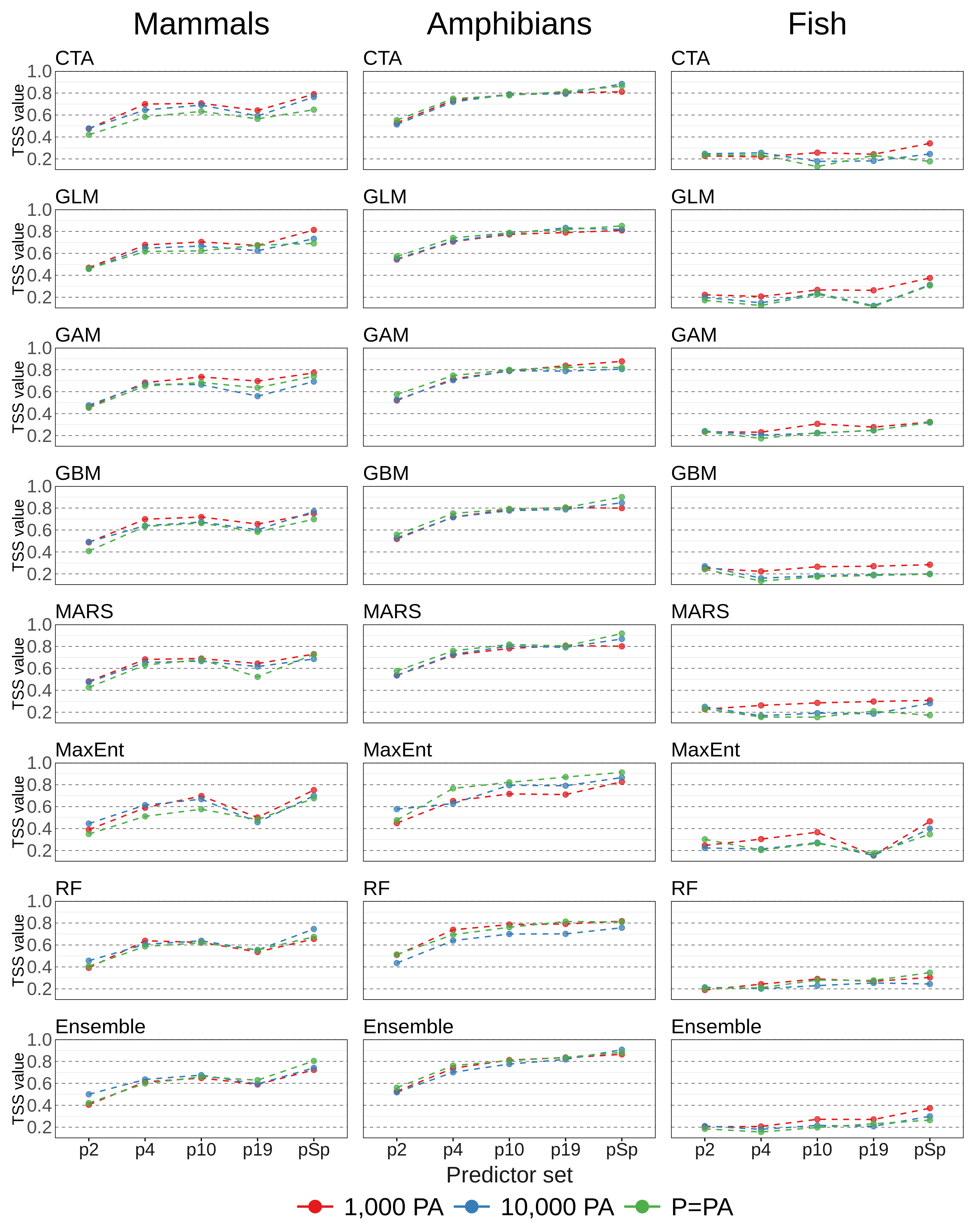


Figure S1: Mean TSS values for each combination of predictor and pseudo-absences selection for each modelling technique in spatially independent testing. Predictor sets include two variables (p2), four variables (p4), a non-redundant set of 10 variables (p10), all 19 bioclimatic variables (p19), and species-specific non-redundant sets (pSp). CTA = Classification Tree Analysis; GAM = Generalized Additive Model; GBM = Generalized Boosted Model; GLM = Generalized Linear Model; MARS = Multivariate Adaptive Regression Splines; MaxEnt = Maximum Entropy; RF = Random Forest.


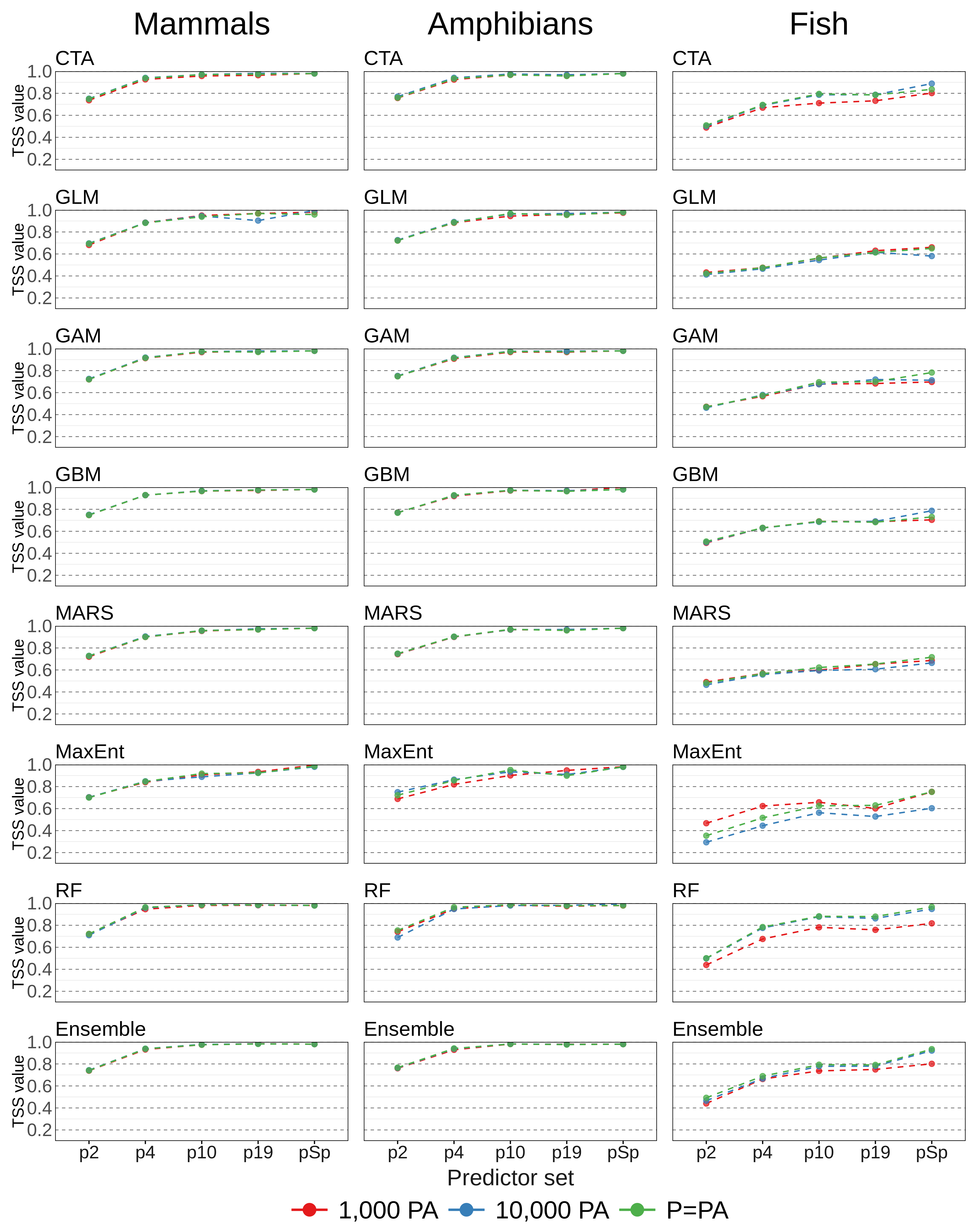
Figure S2: Mean TSS values for each combination of predictor and pseudo-absences selection for each modelling technique in cross-validation. Predictor sets include two variables (p2), four variables (p4), a non-redundant set of 10 variables (p10), all 19 bioclimatic variables (p19), and species-specific non-redundant sets (pSp). CTA = Classification Tree Analysis; GAM = Generalized Additive Model; GBM = Generalized Boosted Model; GLM = Generalized Linear Model; MARS = Multivariate Adaptive Regression Splines; MaxEnt = Maximum Entropy; RF = Random Forest.


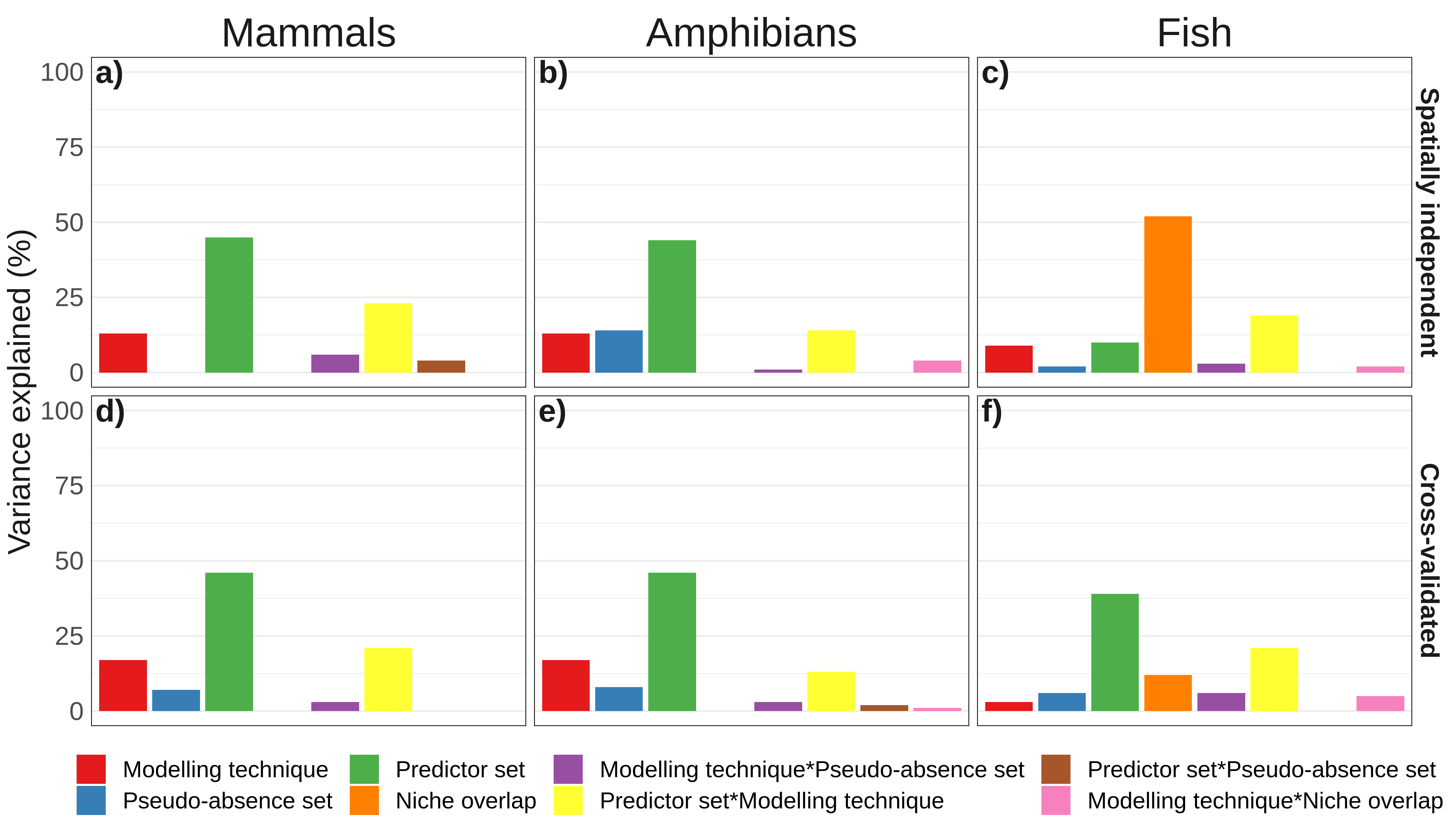
Figure S3: Proportions of variance explained in model performance by predictor set, modelling technique, number of pseudo-absences and niche overlap, for spatially independent testing and cross-validation, excluding the predictor sets with 2 and 19 variables. Proportions of explained variance are given for Mammals (panels a and c), Amphibians (panels b and e), and Fish (panels c and f), both in spatially independent testing (panels a-c) and cross-validation (panels d-f). Proportions of variance explained were rescaled to sum to 100% across the factors.


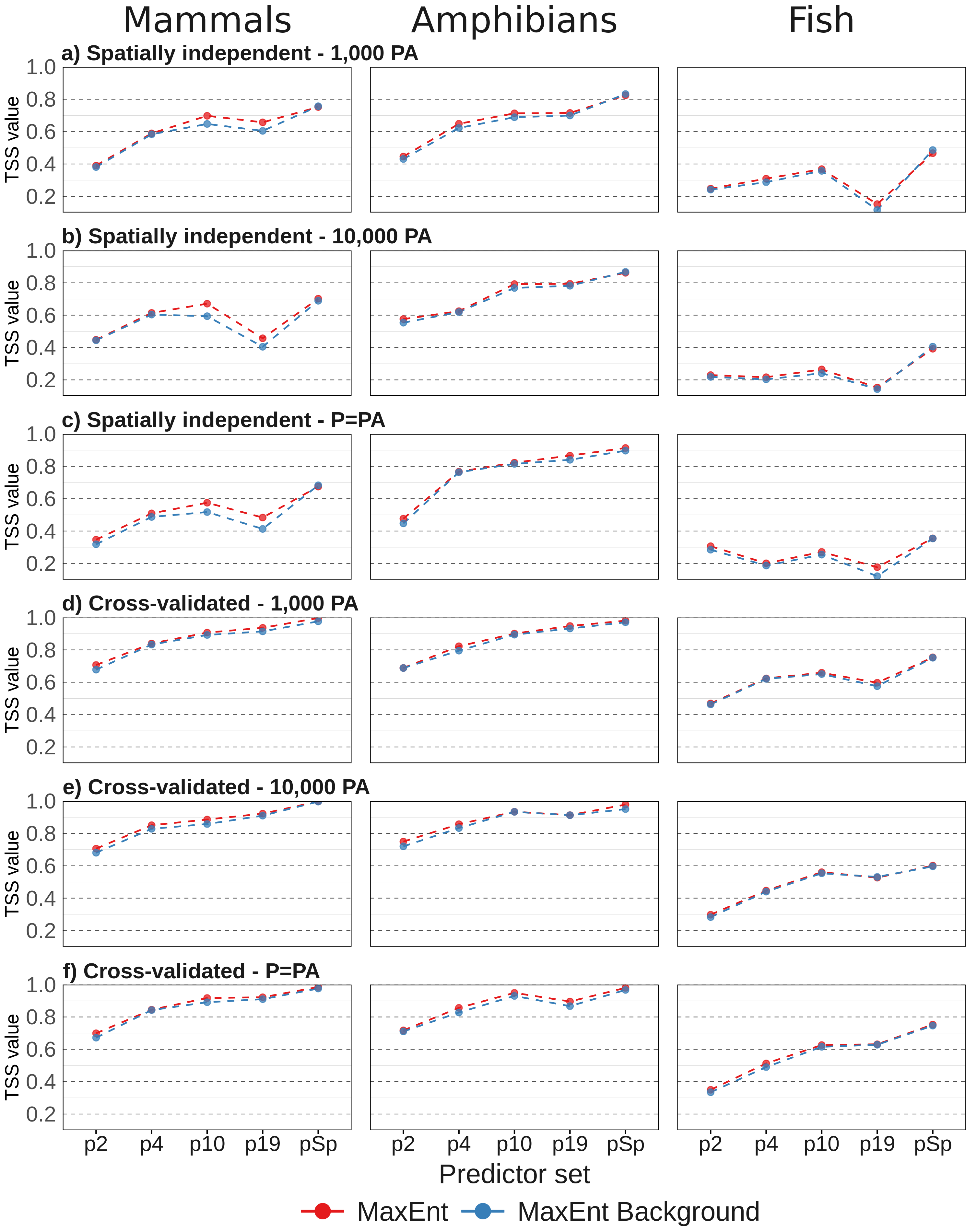
Figure S4: Mean TSS values for each combination of predictor set and MaxEnt and MaxEnt with background data in spatially independent testing and in cross-validation. The results are given for all three pseudo-absence (PA) datasets, including 1,000 PA (panels a and d), 10,000 PA (panels b and e) and a number equal to the number presences (P=PA, panels c and f). Predictor sets include two variables (p2), four variables (p4), a non-redundant set of 10 variables (p10), all 19 bioclimatic variables (p19), and species-specific non-redundant sets (pSp).


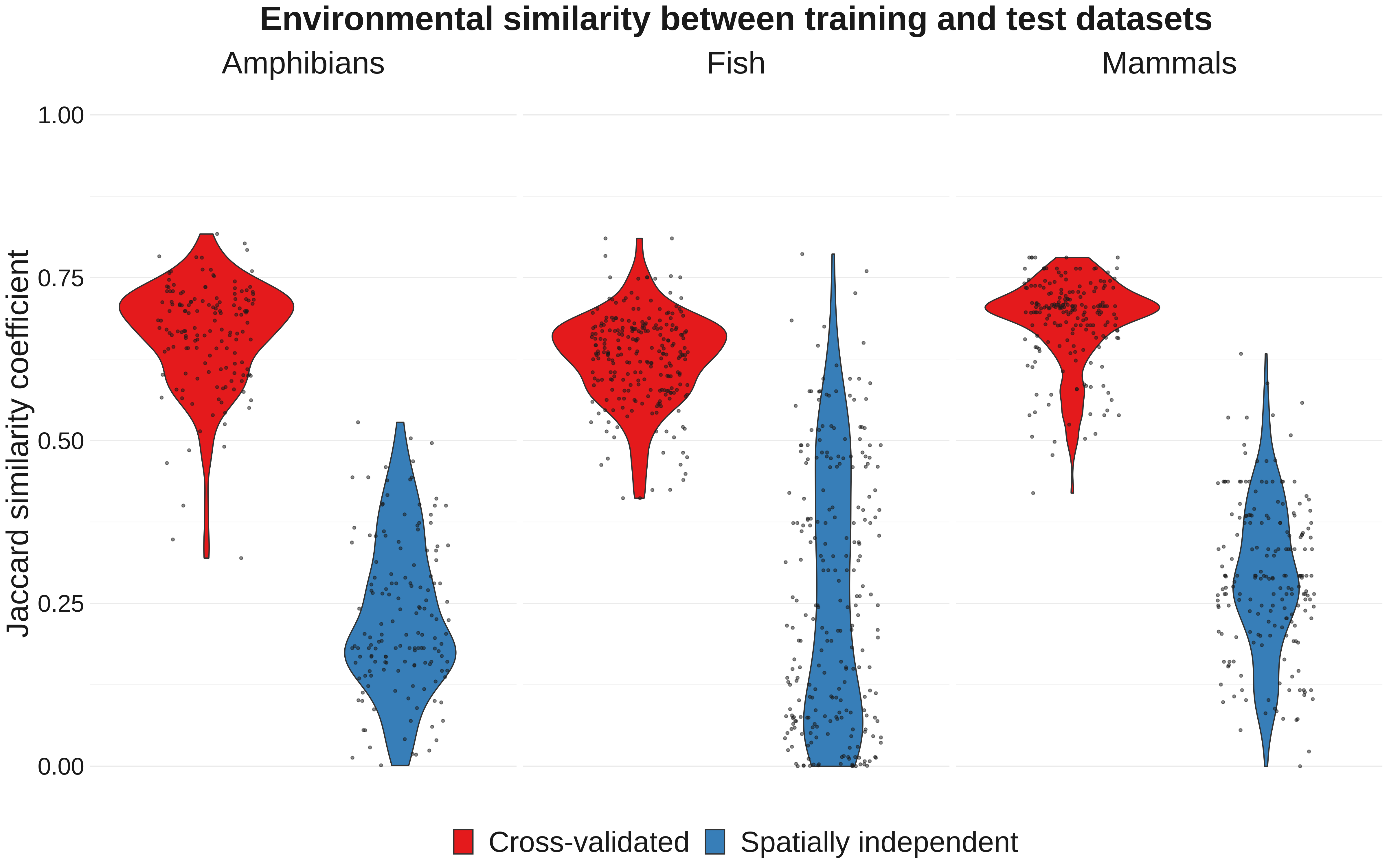
Figure S5: Distribution of environmental overlap values between the training and test datasets, measured by the Jaccard index, for 100 species of amphibians, fish, and mammals. Environmental similarity was determined based on the predictor set with four bioclimatic predictors.

Table S1: List of 100 species per group that were considered for modelling, with the number of variables that remained after stepwise elimination of variables with a VIF threshold of 10. The VIF-based predictor selection was based on the species data including an equal number of pseudo-absences as presences.

| **Species name (amphibians)** | **Number of variables** | **Species name (fish)** | **Number of variables** | **Species name (mammals)** | **Number of variables** |
| --- | --- | --- | --- | --- | --- |
| *Afrixalus dorsalis* | 9 | *Acantopsis choirorhynchos* | 8 | *Abrothrix andinus* | 9 |
| *Ambystoma californiense* | 9 | *Achondrostoma oligolepis* | 8 | *Acomys mullah* | 9 |
| *Ambystoma laterale* | 9 | *Acipenser persicus* | 8 | *Aethomys hindei* | 9 |
| *Ambystoma talpoideum* | 9 | *Alburnoides rossicus* | 8 | *Ailurus fulgens* | 10 |
| *Ameerega hahneli* | 9 | *Alburnus sarmaticus* | 8 | *Akodon albiventer* | 10 |
| *Amolops gerbillus* | 9 | *Ammocrypta clara* | 8 | *Akodon varius* | 10 |
| *Amphiuma means* | 9 | *Anchoviella juruasanga* | 9 | *Allactaga balikunica* | 10 |
| *Anaxyrus americanus* | 9 | *Andersonia leptura* | 7 | *Allactaga hotsoni* | 10 |
| *Anaxyrus californicus* | 9 | *Anguilla bicolor* | 7 | *Alticola argentatus* | 10 |
| *Anaxyrus microscaphus* | 9 | *Aphredoderus sayanus* | 7 | *Alticola roylei* | 9 |
| *Anomaloglossus baeobatrachus* | 9 | *Astyanax guaporensis* | 9 | *Alticola tuvinicus* | 9 |
| *Argenteohyla siemersi* | 9 | *Awaous acritosus* | 9 | *Anomalurus pelii* | 9 |
| *Arthroleptis adelphus* | 9 | *Awaous aeneofuscus* | 9 | *Anoura cultrata* | 9 |
| *Ascaphus montanus* | 8 | *Barbatula altayensis* | 9 | *Anoura latidens* | 9 |
| *Bolitoglossa striatula* | 8 | *Barbichthys laevis* | 8 | *Anoura luismanueli* | 9 |
| *Bombina bombina* | 8 | *Barbus cyclolepis* | 8 | *Aotus lemurinus* | 9 |
| *Breviceps poweri* | 10 | *Batasio dayi* | 8 | *Apodemus agrarius* | 9 |
| *Buergeria buergeri* | 10 | *Brachyplatystoma rousseauxii* | 9 | *Apodemus hyrcanicus* | 9 |
| *Bufotes viridis* | 10 | *Brycon polylepis* | 9 | *Apodemus uralensis* | 9 |
| *Cacosternum karooicum* | 10 | *Capoetobrama kuschakewitschi* | 9 | *Artibeus concolor* | 9 |
| *Cacosternum parvum* | 10 | *Chasmistes cujus* | 9 | *Artibeus lituratus* | 10 |
| *Chiasmocleis leucosticta* | 10 | *Chiloglanis pretoriae* | 9 | *Arvicanthis abyssinicus* | 10 |
| *Dendropsophus soaresi* | 10 | *Chrysichthys johnelsi* | 9 | *Balantiopteryx plicata* | 10 |
| *Duttaphrynus dhufarensis* | 10 | *Clarias angolensis* | 10 | *Bdeogale jacksoni* | 10 |
| *Dyscophus insularis* | 10 | *Clarias buettikoferi* | 10 | *Berylmys berdmorei* | 10 |
| *Eleutherodactylus pipilans* | 10 | *Clarias fuscus* | 7 | *Bos gaurus* | 10 |
| *Espadarana prosoblepon* | 9 | *Clarias werneri* | 8 | *Callosciurus notatus* | 9 |
| *Eurycea lucifuga* | 9 | *Cottus carolinae* | 8 | *Calomys callidus* | 10 |
| *Exerodonta sumichrasti* | 9 | *Cymatogaster aggregata* | 7 | *Calomys sorellus* | 10 |
| *Fejervarya pierrei* | 9 | *Distichodus decemmaculatus* | 7 | *Calomyscus hotsoni* | 10 |
| *Gastrotheca fissipes* | 10 | *Enteromius ablabes* | 10 | *Caluromys philander* | 10 |
| *Hoplobatrachus crassus* | 10 | *Enteromius aspilus* | 10 | *Caracal caracal* | 9 |
| *Hoplobatrachus occipitalis* | 10 | *Enteromius chlorotaenia* | 9 | *Cardioderma cor* | 9 |
| *Hyperolius schoutedeni* | 10 | *Enteromius eutaenia* | 9 | *Caryomys eva* | 10 |
| *Hypsiboas albopunctatus* | 10 | *Enteromius guirali* | 9 | *Cephalophus niger* | 10 |
| *Hypsiboas marianitae* | 10 | *Enteromius kerstenii* | 9 | *Cephalophus rufilatus* | 10 |
| *Ichthyophis nguyenorum* | 9 | *Enteromius kessleri* | 9 | *Cercopithecus neglectus* | 9 |
| *Lechriodus melanopyga* | 9 | *Enteromius macrops* | 9 | *Chaerephon aloysiisabaudiae* | 9 |
| *Leptopelis aubryi* | 9 | *Enteromius miolepis* | 9 | *Chaerephon nigeriae* | 10 |
| *Leptopelis boulengeri* | 10 | *Enteromius unitaeniatus* | 9 | *Chaerephon plicatus* | 10 |
| *Limnodynastes convexiusculus* | 10 | *Enteromius viviparus* | 7 | *Chaetodipus formosus* | 10 |
| *Limnodynastes tasmaniensis* | 10 | *Etheostoma lynceum* | 6 | *Chaetodipus nelsoni* | 10 |
| *Limnonectes poilani* | 10 | *Ethmalosa fimbriata* | 9 | *Choeroniscus minor* | 9 |
| *Lithobates areolatus* | 10 | *Gerres filamentosus* | 9 | *Connochaetes gnou* | 9 |
| *Lithobates capito* | 10 | *Gila elegans* | 9 | *Cremnomys cutchicus* | 9 |
| *Lithobates catesbeianus* | 10 | *Glossogobius callidus* | 8 | *Cricetulus barabensis* | 9 |
| *Litoria caerulea* | 10 | *Gnathonemus petersii* | 8 | *Cricetus cricetus* | 9 |
| *Litoria revelata* | 10 | *Gymnostomus caudimaculatus* | 8 | *Ctenomys magellanicus* | 9 |
| *Litoria rothii* | 10 | *Gymnostomus lineatus* | 8 | *Cuniculus taczanowskii* | 9 |
| *Litoria wilcoxii* | 9 | *Gymnothorax polyuranodon* | 8 | *Cynopterus sphinx* | 10 |
| *Melanophryniscus fulvoguttatus* | 9 | *Kneria polli* | 8 | *Dasypus kappleri* | 9 |
| *Nanorana annandalii* | 9 | *Knipowitschia caucasica* | 8 | *Dasypus sabanicola* | 10 |
| *Necturus punctatus* | 9 | *Kuhlia mugil* | 8 | *Daubentonia madagascariensis* | 10 |
| *Notaden melanoscaphus* | 9 | *Labeo bata* | 8 | *Delomys dorsalis* | 10 |
| *Nyctimystes pulcher* | 10 | *Labeo indramontri* | 8 | *Dendrohyrax arboreus* | 10 |
| *Occidozyga magnapustulosa* | 10 | *Labeo simpsoni* | 9 | *Dendromus melanotis* | 10 |
| *Oreolalax popei* | 10 | *Labeobarbus codringtonii* | 7 | *Dendromus mystacalis* | 10 |
| *Pachytriton brevipes* | 10 | *Leptocypris niloticus* | 7 | *Desmodilliscus braueri* | 9 |
| *Phasmahyla cochranae* | 9 | *Leuciscus aspius* | 7 | *Desmomys harringtoni* | 10 |
| *Phrynobatrachus cornutus* | 9 | *Luciobrama macrocephalus* | 7 | *Dipodomys compactus* | 10 |
| *Phyllodytes acuminatus* | 10 | *Macrognathus siamensis* | 8 | *Dipodomys spectabilis* | 10 |
| *Physalaemus biligonigerus* | 10 | *Microgadus tomcod* | 8 | *Distoechurus pennatus* | 10 |
| *Physalaemus gracilis* | 9 | *Mochokus niloticus* | 8 | *Dolichotis salinicola* | 10 |
| *Plethodon cinereus* | 9 | *Moxostoma lachneri* | 8 | *Dremomys lokriah* | 10 |
| *Pleurodema borellii* | 9 | *Nemacheilus doonensis* | 8 | *Dremomys pernyi* | 10 |
| *Pleurodema cinereum* | 9 | *Neolissochilus baoshanensis* | 8 | *Ectophylla alba* | 8 |
| *Pleurodema nebulosum* | 10 | *Ninnigobius canestrinii* | 8 | *Eliomys quercinus* | 8 |
| *Pristimantis toftae* | 10 | *Noturus flavus* | 9 | *Eliurus myoxinus* | 10 |
| *Pseudacris cadaverina* | 10 | *Oreoglanis setiger* | 7 | *Ellobius fuscocapillus* | 10 |
| *Pseudis tocantins* | 10 | *Oryzias sinensis* | 7 | *Ellobius tancrei* | 10 |
| *Pseudophryne raveni* | 10 | *Pangasius conchophilus* | 8 | *Eolagurus przewalskii* | 10 |
| *Pseudotriton ruber* | 10 | *Parachela siamensis* | 8 | *Eothenomys melanogaster* | 10 |
| *Ptychadena anchietae* | 10 | *Paralaubuca harmandi* | 6 | *Eozapus setchuanus* | 10 |
| *Ptychadena neumanni* | 10 | *Pardiglanis tarabinii* | 9 | *Epomops franqueti* | 10 |
| *Ptychadena oxyrhynchus* | 10 | *Pareuchiloglanis feae* | 9 | *Eptesicus bottae* | 9 |
| *Ptychadena porosissima* | 10 | *Pareutropius debauwi* | 9 | *Eptesicus hottentotus* | 10 |
| *Ptychadena superciliaris* | 9 | *Petrocephalus simus* | 8 | *Erythrocebus patas* | 10 |
| *Rana graeca* | 9 | *Phalacronotus bleekeri* | 8 | *Eumops perotis* | 10 |
| *Rhacophorus annamensis* | 9 | *Pimephales vigilax* | 8 | *Euneomys petersoni* | 10 |
| *Rhacophorus orlovi* | 9 | *Pollimyrus nigricans* | 9 | *Funisciurus leucogenys* | 10 |
| *Rhinella marina* | 9 | *Polypterus weeksii* | 9 | *Funisciurus pyrropus* | 10 |
| *Rhinella veraguensis* | 9 | *Pristigaster whiteheadi* | 9 | *Galago gallarum* | 8 |
| *Salamandra salamandra* | 9 | *Pristolepis fasciata* | 7 | *Gazella dorcas* | 8 |
| *Scaphiopus hurterii* | 9 | *Puntioplites proctozystron* | 7 | *Gazella subgutturosa* | 8 |
| *Scinax castroviejoi* | 9 | *Ramnogaster arcuata* | 8 | *Genetta abyssinica* | 8 |
| *Scinax cruentommus* | 9 | *Ramnogaster melanostoma* | 8 | *Gerbillus dasyurus* | 8 |
| *Scinax fuscomarginatus* | 10 | *Redigobius bikolanus* | 8 | *Gerbillus mackilligini* | 9 |
| *Sclerophrys asmarae* | 10 | *Rhinosardinia bahiensis* | 8 | *Gerbillus somalicus* | 9 |
| *Sclerophrys mauritanica* | 10 | *Romanogobio vladykovi* | 8 | *Glischropus tylopus* | 9 |
| *Scotobleps gabonicus* | 11 | *Sarotherodon caudomarginatus* | 8 | *Gracilinanus emiliae* | 9 |
| *Smilisca fodiens* | 11 | *Serranochromis jallae* | 8 | *Grammomys buntingi* | 9 |
| *Taricha sierra* | 11 | *Serranochromis robustus* | 8 | *Grammomys ibeanus* | 9 |
| *Telmatobius arequipensis* | 11 | *Syngnathus typhle* | 8 | *Grammomys macmillani* | 9 |
| *Telmatobius marmoratus* | 11 | *Synodontis afrofischeri* | 8 | *Graphiurus platyops* | 9 |
| *Trachycephalus jordani* | 11 | *Synodontis notatus* | 8 | *Murexia melanurus* | 9 |
| *Triturus carnifex* | 11 | *Terapon jarbua* | 10 | *Necromys obscurus* | 9 |
| *Triturus karelinii* | 11 | *Tetraodon miurus* | 9 | *Necromys temchuki* | 9 |
| *Uperodon globulosus* | 10 | *Trachelyopterus albicrux* | 8 | *Phyllomys dasythrix* | 9 |
| *Uperoleia fusca* | 10 | *Trichopodus trichopterus* | 8 | *Phyllomys nigrispinus* | 9 |
| *Xenopus epitropicalis* | 9 | *Yasuhikotakia morleti* | 8 | *Vespadelus pumilus* | 9 |

Table S2: Model performance measured by mean TSS value in spatially independent testing for each combination of modelling technique, predictor set and pseudo-absences selection. Values represent means across the species with in brackets the 5 and 95 percentiles. The results are given for all three pseudo-absence sets, including 1,000 PA, 10,000 PA and a number equal to the number of presences (# presences). Predictor sets include two variables (p2), four variables (p4), a non-redundant set of 10 variables (p10), all 19 bioclimatic variables (p19), and species-specific non-redundant sets (pSp). CTA = Classification Tree Analysis; GAM = Generalized Additive Model; GBM = Generalized Boosted Model; GLM = Generalized Linear Model; MARS = Multivariate Adaptive Regression Splines; MaxEnt = Maximum Entropy; RF = Random Forest.

| **Taxonomic group** | **Pseudo-absence set** | **Predictor set** | **Modelling technique** | | | | | | |  | |
| --- | --- | --- | --- | --- | --- | --- | --- | --- | --- | --- | --- |
|  |  |  | **CTA** | **GAM** | **GBM** | **GLM** | **MARS** | **MaxEnt** | **RF** | | **Ensemble** |
| Mammals | 10,000 | p2 | 0.48 (0.1, 0.88) | 0.5 (0.05, 0.89) | 0.49 (0.1, 0.9) | 0.47 (0.03, 0.86) | 0.49 (0.03, 0.91) | 0.48 (0.05, 0.86) | 0.46 (0.12, 0.85) | | 0.5 (0.06, 0.89) |
|  |  | p4 | 0.7 (0.3, 0.98) | 0.7 (0.37, 0.98) | 0.7 (0.27, 0.98) | 0.67 (0.34, 0.99) | 0.7 (0.35, 0.99) | 0.58 (0.19, 0.91) | 0.66 (0.24, 0.98) | | 0.7 (0.28, 0.98) |
|  |  | p10 | 0.75 (0.3, 0.98) | 0.77 (0.42, 0.98) | 0.75 (0.28, 0.99) | 0.76 (0.16, 0.99) | 0.76 (0.32, 0.99) | 0.67 (0.21, 0.95) | 0.72 (0.29, 0.98) | | 0.77 (0.02, 0.97) |
|  |  | p19 | 0.81 (0.46, 0.99) | 0.81 (0.45, 0.98) | 0.78 (0.38, 0.99) | 0.8 (0.51, 0.98) | 0.81 (0.54, 0.99) | 0.68 (0.1, 0.97) | 0.73 (0.37, 0.99) | | 0.8 (0.05, 0.98) |
|  |  | pSp | 0.85 (0.3, 0.98) | 0.82 (0.46, 0.98) | 0.82 (0.33, 0.99) | 0.76 (0.16, 0.99) | 0.81 (0.36, 0.99) | 0.73 (0.28, 0.99) | 0.78 (0.33, 0.98) | | 0.84 (0.15, 0.97) |
|  |  |  |  |  |  |  |  |  |  | |  |
|  | # presences | p2 | 0.5 (0.03, 0.94) | 0.5 (-0.04, 0.92) | 0.5 (-0.02, 0.94) | 0.45 (0, 0.91) | 0.49 (0.00, 0.92) | 0.47 (-0.12, 0.86) | 0.5 (0.09, 0.87) | | 0.51 (0.02, 0.94) |
|  |  | p4 | 0.71 (0.2, 0.98) | 0.73 (0.37, 0.98) | 0.71 (0.27, 0.99) | 0.7 (0.26, 0.99) | 0.73 (0.31, 0.98) | 0.61 (0.22, 0.93) | 0.71 (0.29, 0.98) | | 0.74 (0.28, 0.99) |
|  |  | p10 | 0.78 (0.47, 0.99) | 0.84 (0.61, 0.98) | 0.8 (0.45, 0.99) | 0.81 (0.51, 0.99) | 0.79 (0.42, 0.99) | 0.65 (0.25, 0.96) | 0.77 (0.41, 0.99) | | 0.82 (0.47, 0.99) |
|  |  | p19 | 0.77 (0.37, 0.99) | 0.81 (0.39, 0.99) | 0.8 (0.26, 0.99) | 0.8 (0.33, 0.99) | 0.79 (0.37, 0.99) | 0.68 (0.27, 0.95) | 0.77 (0.38, 0.99) | | 0.81 (0.46, 0.99) |
|  |  | pSp | 0.83 (0.3, 0.98) | 0.86 (0.56, 0.98) | 0.82 (0.33, 0.99) | 0.86 (0.36, 0.99) | 0.83 (0.41, 0.99) | 0.73 (0.26, 0.98) | 0.79 (0.43, 0.98) | | 0.89 (0.45, 0.99) |
|  |  |  |  |  |  |  |  |  |  | |  |
|  | 1,000 | p2 | 0.49 (0.06, 0.9) | 0.47 (0, 0.89) | 0.5 (0.04, 0.9) | 0.48 (-0.02, 0.86) | 0.49 (0.09, 0.89) | 0.46 (0.06, 0.86) | 0.52 (0.11, 0.86) | | 0.52 (0.1, 0.9) |
|  |  | p4 | 0.68 (0.35, 0.98) | 0.66 (0.36, 0.97) | 0.69 (0.29, 0.98) | 0.66 (0.26, 0.98) | 0.67 (0.3, 0.95) | 0.56 (0.14, 0.9) | 0.76 (0.34, 0.98) | | 0.71 (0.33, 0.98) |
|  |  | p10 | 0.74 (0.21, 0.98) | 0.79 (0.34, 0.99) | 0.76 (0.38, 0.99) | 0.74 (0.32, 0.98) | 0.72 (0.3, 0.98) | 0.66 (0.31, 0.96) | 0.81 (0.41, 0.99) | | 0.79 (0.36, 0.99) |
|  |  | p19 | 0.76 (0.24, 0.99) | 0.8 (0.34, 0.98) | 0.78 (0.26, 0.99) | 0.78 (0.35, 0.98) | 0.79 (0.43, 0.97) | 0.68 (0.16, 0.97) | 0.8 (0.34, 0.99) | | 0.82 (0.34, 0.99) |
|  |  | pSp | 0.83 (0.32, 0.98) | 0.84 (0.46, 0.98) | 0.82 (0.31, 0.99) | 0.82 (0.38, 0.99) | 0.81 (0.41, 0.99) | 0.77 (0.21, 0.97) | 0.84 (0.41, 0.99) | | 0.86 (0.42, 0.99) |
|  |  |  |  |  |  |  |  |  |  | |  |
| Amphibians | 10,000 | p2 | 0.49 (0.02, 0.9) | 0.49 (0.07, 0.91) | 0.49 (0.02, 0.9) | 0.52 (0.05, 0.92) | 0.51 (0.12, 0.91) | 0.47 (0.09, 0.91) | 0.41 (0.02, 0.85) | | 0.49 (0.06, 0.9) |
|  |  | p4 | 0.72 (0.31, 0.98) | 0.68 (0.29, 0.98) | 0.72 (0.32, 0.98) | 0.7 (0.29, 0.99) | 0.73 (0.38, 0.97) | 0.65 (0.26, 0.89) | 0.62 (0.17, 0.97) | | 0.7 (0.36, 0.98) |
|  |  | p10 | 0.79 (0.44, 0.99) | 0.74 (0.28, 0.99) | 0.77 (0.38, 0.98) | 0.77 (0.39, 0.99) | 0.78 (0.45, 0.99) | 0.63 (0.22, 0.91) | 0.68 (0.3, 0.98) | | 0.76 (0.39, 0.99) |
|  |  | p19 | 0.79 (0.43, 0.99) | 0.73 (0.32, 0.99) | 0.77 (0.37, 0.99) | 0.82 (0.45, 0.99) | 0.76 (0.26, 0.99) | 0.7 (0.17, 0.95) | 0.67 (0.26, 0.99) | | 0.78 (0.38, 0.99) |
|  |  | pSp | 0.81 (0.42, 0.98) | 0.76 (0.36, 0.98) | 0.81 (0.37, 0.99) | 0.84 (0.46, 0.99) | 0.81 (0.40, 0.99) | 0.75 (0.25, 0.98) | 0.74 (0.41, 0.99) | | 0.86 (0.42, 0.99) |
|  |  |  |  |  |  |  |  |  |  | |  |
|  | # presences | p2 | 0.5 (0, 0.89) | 0.52 (0.05, 0.92) | 0.51 (-0.01, 0.9) | 0.53 (-0.02, 0.92) | 0.54 (0.12, 0.92) | 0.48 (0.04, 0.89) | 0.45 (0.01, 0.84) | | 0.51 (0.02, 0.92) |
|  |  | p4 | 0.75 (0.33, 0.98) | 0.72 (0.3, 0.98) | 0.75 (0.34, 0.99) | 0.73 (0.31, 0.98) | 0.76 (0.41, 0.97) | 0.71 (0.4, 0.98) | 0.67 (0.3, 0.98) | | 0.75 (0.36, 0.98) |
|  |  | p10 | 0.77 (0.42, 0.98) | 0.76 (0.31, 0.99) | 0.79 (0.38, 0.99) | 0.78 (0.3, 0.99) | 0.79 (0.41, 0.99) | 0.68 (0.16, 0.97) | 0.74 (0.4, 0.99) | | 0.78 (0.35, 1) |
|  |  | p19 | 0.81 (0.45, 0.98) | 0.77 (0.43, 0.98) | 0.81 (0.37, 0.99) | 0.78 (0.09, 1) | 0.8 (0.48, 0.99) | 0.75 (0.29, 0.98) | 0.74 (0.32, 0.99) | | 0.81 (0.46, 1) |
|  |  | pSp | 0.83 (0.3, 0.98) | 0.86 (0.56, 0.98) | 0.82 (0.33, 0.99) | 0.86 (0.36, 0.99) | 0.83 (0.41, 0.99) | 0.73 (0.26, 0.98) | 0.79 (0.43, 0.98) | | 0.89 (0.45, 0.99) |
|  |  |  |  |  |  |  |  |  |  | |  |
|  | 1,000 | p2 | 0.5 (0.02, 0.91) | 0.47 (-0.06, 0.92) | 0.49 (0, 0.92) | 0.51 (0.01, 0.93) | 0.51 (0.15, 0.9) | 0.5 (0.09, 0.91) | 0.47 (0.03, 0.88) | | 0.49 (0.04, 0.91) |
|  |  | p4 | 0.73 (0.33, 0.98) | 0.69 (0.24, 0.98) | 0.72 (0.31, 0.98) | 0.7 (0.29, 0.99) | 0.72 (0.32, 0.97) | 0.61 (0.17, 0.97) | 0.71 (0.32, 0.99) | | 0.73 (0.34, 0.99) |
|  |  | p10 | 0.76 (0.43, 0.99) | 0.75 (0.41, 0.98) | 0.78 (0.38, 0.98) | 0.76 (0.31, 0.99) | 0.78 (0.43, 0.98) | 0.7 (0.18, 0.97) | 0.75 (0.39, 0.99) | | 0.8 (0.44, 0.99) |
|  |  | p19 | 0.77 (0.4, 0.99) | 0.8 (0.41, 0.98) | 0.8 (0.45, 0.99) | 0.76 (0.39, 0.99) | 0.78 (0.4, 0.98) | 0.69 (0.11, 0.95) | 0.73 (0.26, 0.99) | | 0.8 (0.38, 1) |
|  |  | pSp | 0.81 (0.42, 0.98) | 0.76 (0.36, 0.98) | 0.81 (0.37, 0.99) | 0.84 (0.46, 0.99) | 0.81 (0.40, 0.99) | 0.75 (0.25, 0.98) | 0.74 (0.41, 0.99) | | 0.86 (0.42, 0.99) |
|  |  |  |  |  |  |  |  |  |  | |  |
| Fishes | 10,000 | p2 | 0.27 (-0.19, 0.61) | 0.28 (-0.26, 0.62) | 0.3 (-0.18, 0.64) | 0.28 (-0.04, 0.64) | 0.28 (-0.05, 0.6) | 0.35 (-0.07, 0.64) | 0.27 (-0.1, 0.55) | | 0.3 (-0.17, 0.63) |
|  |  | p4 | 0.36 (-0.07, 0.78) | 0.36 (-0.21, 0.86) | 0.36 (-0.32, 0.82) | 0.32 (-0.06, 0.74) | 0.29 (-0.36, 0.75) | 0.28 (-0.55, 0.8) | 0.37 (-0.06, 0.73) | | 0.38 (-0.12, 0.8) |
|  |  | p10 | 0.39 (-0.15, 0.86) | 0.39 (-0.21, 0.85) | 0.41 (-0.1, 0.87) | 0.4 (0.04, 0.81) | 0.43 (-0.06, 0.88) | 0.37 (-0.25, 0.86) | 0.41 (0.01, 0.84) | | 0.42 (-0.08, 0.89) |
|  |  | p19 | 0.35 (-0.09, 0.81) | 0.38 (-0.08, 0.83) | 0.4 (-0.12, 0.85) | 0.34 (-0.25, 0.79) | 0.42 (-0.04, 0.86) | 0.38 (-0.07, 0.85) | 0.42 (0.03, 0.84) | | 0.45 (-0.07, 0.9) |
|  |  | pSp | 0.43 (0.12, 0.88) | 0.44 (0.16, 0.88) | 0.42 (0.01, 0.89) | 0.42 (0.08, 0.83) | 0.41 (0.01, 0.89) | 0.47 (0.01, 0.87) | 0.44 (-0.01, 0.89) | | 0.46 (0.12, 0.91) |
|  |  |  |  |  |  |  |  |  |  | |  |
|  | # presences | p2 | 0.23 (-0.23, 0.6) | 0.25 (-0.33, 0.63) | 0.25 (-0.22, 0.62) | 0.25 (0.01, 0.56) | 0.26 (-0.1, 0.6) | 0.3 (-0.02, 0.62) | 0.27 (-0.12, 0.58) | | 0.27 (-0.22, 0.59) |
|  |  | p4 | 0.34 (-0.17, 0.72) | 0.31 (-0.23, 0.87) | 0.32 (-0.32, 0.84) | 0.31 (-0.08, 0.78) | 0.31 (-0.23, 0.81) | 0.29 (-0.39, 0.77) | 0.37 (-0.11, 0.73) | | 0.35 (-0.14, 0.84) |
|  |  | p10 | 0.36 (-0.15, 0.82) | 0.37 (-0.22, 0.85) | 0.39 (-0.1, 0.86) | 0.39 (0.02, 0.89) | 0.41 (-0.1, 0.81) | 0.36 (-0.1, 0.91) | 0.44 (0.01, 0.9) | | 0.44 (-0.01, 0.88) |
|  |  | p19 | 0.35 (-0.12, 0.77) | 0.39 (-0.05, 0.82) | 0.43 (0, 0.9) | 0.36 (-0.25, 0.81) | 0.45 (0.03, 0.86) | 0.41 (-0.05, 0.84) | 0.47 (0.03, 0.92) | | 0.47 (-0.01, 0.91) |
|  |  | pSp | 0.42 (0.11, 0.88) | 0.41 (0.06, 0.88) | 0.46 (0.01, 0.89) | 0.48 (0.02, 0.81) | 0.41 (0.01, 0.85) | 0.43 (0.01, 0.87) | 0.48 (-0.01, 0.89) | | 0.46 (0.06, 0.91) |
|  |  |  |  |  |  |  |  |  |  | |  |
|  | 1,000 | p2 | 0.23 (-0.2, 0.67) | 0.23 (-0.32, 0.69) | 0.26 (-0.18, 0.67) | 0.22 (-0.39, 0.65) | 0.22 (-0.31, 0.59) | 0.23 (-0.15, 0.64) | 0.26 (-0.13, 0.67) | | 0.25 (-0.27, 0.62) |
|  |  | p4 | 0.28 (-0.36, 0.77) | 0.27 (-0.22, 0.7) | 0.28 (-0.28, 075) | 0.27 (-0.12, 0.68) | 0.26 (-0.2, 0.65) | 0.28 (-0.3, 0.78) | 0.38 (-0.16, 0.86) | | 0.32 (-0.26, 0.8) |
|  |  | p10 | 0.34 (-0.12, 0.82) | 0.34 (-0.12, 0.82) | 0.37 (-0.14, 0.87) | 0.37 (-0.06, 0.8) | 0.34 (-0.08, 0.87) | 0.32 (-0.25, 0.75) | 0.48 (-0.06, 0.98) | | 0.41 (-0.04, 0.9) |
|  |  | p19 | 0.33 (0.11, 0.86) | 0.39 (0.06, 0.85) | 0.42 (0.01, 0.87) | 0.41 (0.08, 0.83) | 0.41 (0.01, 0.89) | 0.37 (0.01, 0.87) | 0.44 (-0.01, 0.89) | | 0.45 (0.12, 0.91) |
|  |  | pSp | 0.35 (-0.12, 0.77) | 0.43 (0.05, 0.82) | 0.43 (0, 0.9) | 0.46 (-0.05, 0.81) | 0.45 (0.13, 0.86) | 0.41 (0.05, 0.84) | 0.47 (0.03, 0.92) | | 0.47 (0.03, 0.91) |
|  |  |  |  |  |  |  |  |  |  | |  |

Table S3: Model performance measured by mean TSS value in cross-validation for each combination of modelling technique, predictor and pseudo-absences selection. Values represent means across the species with in between brackets the 5 and 95 percentiles. The results are given for all three pseudo-absence sets, including 1,000 PA, 10,000 PA and a number equal to the number of presences (# presences). Predictor sets include two variables (p2), four variables (p4), a non-redundant set of 10 variables (p10), all 19 bioclimatic variables (p19), and species-specific non-redundant sets (pSp). CTA = Classification Tree Analysis; GAM = Generalized Additive Model; GBM = Generalized Boosted Model; GLM = Generalized Linear Model; MARS = Multivariate Adaptive Regression Splines; MaxEnt = Maximum Entropy; RF = Random Forest.

| **Taxonomic group** | **Pseudo-absence set** | **Predictor set** | **Modelling technique** | | | | | | |  | |
| --- | --- | --- | --- | --- | --- | --- | --- | --- | --- | --- | --- |
|  |  |  | **CTA** | **GAM** | **GBM** | **GLM** | **MARS** | **MaxEnt** | **RF** | | **Ensemble** |
| Mammals | 10,000 | p2 | 0.73 (0.49, 0.93) | 0.7 (0.44, 0.93) | 0.73 (0.49, 0.94) | 0.67 (0.37, 0.92) | 0.7 (0.43, 0.92) | 0.68 (0.43, 0.91) | 0.7 (0.41, 0.93) | | 0.72 (0.47, 0.94) |
|  |  | p4 | 0.92 (0.81, 0.99) | 0.89 (0.7, 0.99) | 0.91 (0.75, 0.99 | 0.85 (0.6, 0.99) | 0.88 (0.66, 0.99) | 0.83 (0.65, 0.94) | 0.95 (0.87, 0.99) | | 0.92 (0.79, 0.99) |
|  |  | p10 | 0.96 (0.89, 0.99) | 0.96 (0.84, 1) | 0.95 (0.85, 0.99) | 0.92 (0.76, 0.99) | 0.94 (0.79, 0.99) | 0.89 (0.79, 0.97) | 0.98 (0.96, 1) | | 0.97 (0.89, 0.99) |
|  |  | p19 | 0.97 (0.89, 0.99) | 0.98 (0.95, 1) | 0.97 (0.92, 1) | 0.95 (0.83, 1) | 0.97 (0.91, 1) | 0.91 (0.81, 0.99) | 0.98 (0.96, 1) | | 0.98 (0.95, 1) |
|  |  | pSp | 0.96 (0.89, 0.99) | 0.96 (0.84, 1) | 0.95 (0.85, 0.99) | 0.92 (0.76, 0.99) | 0.94 (0.79, 0.99) | 0.89 (0.79, 0.97) | 0.98 (0.96, 1) | | 0.97 (0.89, 0.99) |
|  |  |  |  |  |  |  |  |  |  | |  |
|  | # presences | p2 | 0.73 (0.49, 0.93) | 0.7 (0.44, 0.93) | 0.73 (0.49, 0.94) | 0.67 (0.37, 0.92) | 0.7 (0.44, 0.92) | 0.67 (0.42, 0.91) | 0.72 (0.48, 0.93) | | 0.72 (0.5, 0.93) |
|  |  | p4 | 0.92 (0.81, 0.99) | 0.89 (0.7, 0.99) | 0.91 (0.75, 0.99) | 0.85 (0.58, 0.99) | 0.88 (0.66, 0.99) | 0.82 (0.67, 0.96) | 0.95 (0.87, 1) | | 0.92 (0.78, 0.99) |
|  |  | p10 | 0.96 (0.9, 0.99) | 0.96 (0.85, 1) | 0.95 (0.85, 1) | 0.93 (0.74, 1) | 0.94 (0.79, 0.99) | 0.89 (0.79, 0.97) | 0.98 (0.96, 1) | | 0.97 (0.9, 1) |
|  |  | p19 | 0.97 (0.92, 1) | 0.97 (0.94, 1) | 0.97 (0.91, 1) | 0.95 (0.86, 1) | 0.95 (0.9, 1) | 0.91 (0.81, 0.98) | 0.98 (0.96, 1) | | 0.98 (0.95, 1) |
|  |  | pSp | 0.97 (0.93, 1) | 0.97 (0.92, 0.99) | 0.97 (0.93, 1) | 0.97 (0.89, 1) | 0.97 (0.9, 1) | 0.91 (0.83, 0.97) | 0.97 (0.93, 1) | | 0.98 (0.95, 1) |
|  |  |  |  |  |  |  |  |  |  | |  |
|  | 1,000 | p2 | 0.72 (0.47, 0.94) | 0.7 (0.53, 0.93) | 0.72 (0.47, 0.93) | 0.66 (0.33, 0.92) | 0.7 (0.45, 0.93) | 0.65 (0.4, 0.9) | 0.69 (0.33, 0.93) | | 0.72 (0.46, 0.93) |
|  |  | p4 | 0.9 (0.78, 0.99) | 0.89 (0.68, 1) | 0.9 (0.73, 1) | 0.85 (0.57, 0.99) | 0.88 (0.7, 0.99) | 0.81 (0.64, 0.96) | 0.93 (0.72, 1) | | 0.91 (0.75, 1) |
|  |  | p10 | 0.95 (0.86, 1) | 0.95 (0.83, 1) | 0.95 (0.86, 1) | 0.93 (0.76, 1) | 0.93 (0.77, 1) | 0.88 (0.76, 0.98) | 0.97 (0.93, 1) | | 0.96 (0.88, 1) |
|  |  | p19 | 0.95 (0.84, 1) | 0.96 (0.86, 1) | 0.96 (0.87, 1) | 0.93 (0.77, 1) | 0.95 (0.85, 1) | 0.91 (0.78, 0.98) | 0.98 (0.93, 1) | | 0.97 (0.91, 1) |
|  |  | pSp | 0.97 (0.89, 0.99) | 0.98 (0.95, 1) | 0.97 (0.92, 1) | 0.95 (0.83, 1) | 0.97 (0.91, 1) | 0.91 (0.81, 0.99) | 0.98 (0.96, 1) | | 0.98 (0.95, 1) |
|  |  |  |  |  |  |  |  |  |  | |  |
| Amphibians | 10,000 | p2 | 0.77 (0.55, 0.93) | 0.75 (0.52, 0.93) | 0.77 (0.54, 0.93) | 0.72 (0.43, 0.92) | 0.74 (0.52, 0.92) | 0.73 (0.47, 0.89) | 0.68 (0.27, 0.92) | | 0.76 (0.52, 0.93) |
|  |  | p4 | 0.94 (0.88, 0.99) | 0.92 (0.81, 0.99) | 0.93 (0.85, 0.99) | 0.9 (0.75, 0.99) | 0.91 (0.78, 0.99) | 0.86 (0.76, 0.94) | 0.95 (0.86, 0.99) | | 0.94 (0.88, 0.99) |
|  |  | p10 | 0.97 (0.93, 1) | 0.97 (0.91, 1) | 0.97 (0.92, 1) | 0.95 (0.75, 1) | 0.96 (0.91, 1) | 0.89 (0.81, 0.95) | 0.97 (0.92, 1) | | 0.98 (0.95, 1) |
|  |  | p19 | 0.97 (0.93, 1) | 0.97 (0.92, 0.99) | 0.97 (0.93, 1) | 0.97 (0.89, 1) | 0.97 (0.9, 1) | 0.91 (0.83, 0.97) | 0.97 (0.93, 1) | | 0.98 (0.95, 1) |
|  |  | pSp | 0.97 (0.92, 1) | 0.97 (0.92, 1) | 0.97 (0.92, 1) | 0.95 (0.9, 1) | 0.97 (0.91, 1) | 0.92 (0.85, 0.98) | 0.98 (0.94, 1) | | 0.98 (0.94, 1) |
|  |  |  |  |  |  |  |  |  |  | |  |
|  | # presences | p2 | 0.76 (0.51, 0.94) | 0.74 (0.5, 0.93) | 0.76 (0.52, 0.93) | 0.72 (0.41, 0.93) | 0.75 (0.5, 0.93) | 0.71 (0.41, 0.88) | 0.74 (0.5, 0.93) | | 0.76 (0.49, 0.94) |
|  |  | p4 | 0.93 (0.84, 0.99) | 0.92 (0.81, 0.99) | 0.93 (0.86, 0.99) | 0.89 (0.76, 0.98) | 0.9 (0.79, 0.99) | 0.86 (0.72, 0.95) | 0.96 (0.91, 1) | | 0.94 (0.87, 1) |
|  |  | p10 | 0.96 (0.92, 0.99) | 0.97 (0.91, 1) | 0.97 (0.93, 1) | 0.95 (0.89, 1) | 0.96 (0.9, 1) | 0.9 (0.82, 0.99) | 0.98 (0.96, 1) | | 0.98 (0.94, 1) |
|  |  | p19 | 0.97 (0.91, 0.99) | 0.97 (0.92, 1) | 0.97 (0.94, 1) | 0.96 (0.88, 1) | 0.97 (0.93, 1) | 0.93 (0.84, 0.99) | 0.98 (0.95, 1) | | 0.98 (0.96, 1) |
|  |  | pSp | 0.95 (0.86, 1) | 0.95 (0.83, 1) | 0.95 (0.86, 1) | 0.93 (0.76, 1) | 0.93 (0.77, 1) | 0.88 (0.76, 0.98) | 0.97 (0.93, 1) | | 0.96 (0.88, 1) |
|  |  |  |  |  |  |  |  |  |  | |  |
|  | 1,000 | p2 | 0.75 (0.52, 0.94) | 0.75 (0.52, 0.94) | 0.76 (0.56, 0.95) | 0.72 (0.43, 0.93) | 0.74 (0.5, 0.94) | 0.72 (0.47, 0.89) | 0.74 (0.49, 0.94) | | 0.76 (0.53, 0.94) |
|  |  | p4 | 0.92 (0.85, 0.99) | 0.91 (0.78, 0.99) | 0.92 (0.83, 0.99) | 0.89 (0.73, 0.99) | 0.9 (0.77, 0.99) | 0.85 (0.72, 0.95) | 0.95 (0.88, 0.99) | | 0.93 (0.85, 1) |
|  |  | p10 | 0.95 (0.91, 1) | 0.96 (0.91, 1) | 0.96 (0.92, 1) | 0.95 (0.83, 1) | 0.96 (0.9, 1) | 0.89 (0.81, 0.98) | 0.98 (0.94, 1) | | 0.97 (0.93, 1) |
|  |  | p19 | 0.96 (0.91, 1) | 0.96 (0.91, 1) | 0.97 (0.92, 1) | 0.95 (0.89, 1) | 0.97 (0.91, 1) | 0.92 (0.81, 0.98) | 0.98 (0.94, 1) | | 0.98 (0.94, 1) |
|  |  | pSp | 0.96 (0.92, 0.99) | 0.97 (0.91, 1) | 0.97 (0.93, 1) | 0.95 (0.89, 1) | 0.96 (0.9, 1) | 0.9 (0.82, 0.99) | 0.98 (0.96, 1) | | 0.98 (0.94, 1) |
|  |  |  |  |  |  |  |  |  |  | |  |
| Fishes | 10,000 | p2 | 0.62 (0.36, 0.85) | 0.56 (0.28, 0.83) | 0.61 (0.34, 0.85) | 0.51 (0.2, 0.81) | 0.57 (0.3, 0.82) | 0.54 (0.2, 0.82) | 0.6 (0.35, 0.82) | | 0.61 (0.34, 0.83) |
|  |  | p4 | 0.8 (0.6, 0.94) | 0.7 (0.44, 0.94) | 0.75 (0.53, 0.93) | 0.62 (0.33, 0.91) | 0.69 (0.43, 0.93) | 0.65 (0.35, 0.89) | 0.86 (0.72, 0.96) | | 0.79 (0.57, 0.94) |
|  |  | p10 | 0.87 (0.72, 0.96) | 0.82 (0.58, 0.96) | 0.82 (0.58, 0.95) | 0.72 (0.44, 0.92) | 0.76 (0.49, 0.94) | 0.72 (0.5, 0.92) | 0.93 (0.84, 0.98) | | 0.87 (0.71, 0.97) |
|  |  | p19 | 0.88 (0.71, 0.96) | 0.87 (0.63, 0.97) | 0.83 (0.59, 0.96) | 0.78 (0.5, 0.96) | 0.8 (0.54, 0.96) | 0.75 (0.51, 0.92) | 0.92 (0.83, 0.98) | | 0.89 (0.73, 0.97) |
|  |  | pSp | 0.95 (0.84, 1) | 0.96 (0.86, 1) | 0.96 (0.87, 1) | 0.93 (0.77, 1) | 0.95 (0.85, 1) | 0.91 (0.78, 0.98) | 0.98 (0.93, 1) | | 0.97 (0.91, 1) |
|  |  |  |  |  |  |  |  |  |  | |  |
|  | # presences | p2 | 0.62 (0.36, 0.85) | 0.56 (0.26, 0.84) | 0.61 (0.36, 0.85) | 0.51 (0.2, 0.82) | 0.57 (0.3, 0.83) | 0.54 (0.21, 0.85) | 0.62 (0.37, 0.83) | | 0.62 (0.36, 0.85) |
|  |  | p4 | 0.8 (0.61, 0.93) | 0.69 (0.45, 0.94) | 0.75 (0.54, 0.93) | 0.61 (0.36, 0.9) | 0.68 (0.42, 0.92) | 0.66 (0.4, 0.88) | 0.87 (0.74, 0.96) | | 0.79 (0.61, 0.94) |
|  |  | p10 | 0.86 (0.72, 0.95) | 0.81 (0.59, 0.96) | 0.82 (0.61, 0.95) | 0.72 (0.44, 0.95) | 0.76 (0.51, 0.95) | 0.73 (0.56, 0.92) | 0.93 (0.85, 0.98) | | 0.87 (0.73, 0.95) |
|  |  | p19 | 0.87 (0.7, 0.96) | 0.86 (0.64, 0.96) | 0.83 (0.6, 0.96) | 0.78 (0.51, 0.96) | 0.8 (0.51, 0.95) | 0.77 (0.56, 0.92) | 0.93 (0.85, 0.98) | | 0.89 (0.74, 0.97) |
|  |  | pSp | 0.92 (0.82, 1) | 0.91 (0.81, 1) | 0.85 (0.71, 1) | 0.83 (0.57, 0.99) | 0.85 (0.65, 0.97) | 0.81 (0.68, 0.98) | 0.98 (0.93, 0.99) | | 0.97 (0.91, 1) |
|  |  |  |  |  |  |  |  |  |  | |  |
|  | 1,000 | p2 | 0.6 (0.34, 0.84) | 0.56 (0.26, 0.84) | 0.6 (0.33, 0.84) | 0.51 (0.22, 0.81) | 0.57 (0.26, 0.83) | 0.52 (0.19, 0.84) | 0.58 (0.22, 0.83) | | 0.59 (0.32, 0.85) |
|  |  | p4 | 0.77 (0.34, 0.84) | 0.69 (0.41, 0.94) | 0.75 (0.49, 0.94) | 0.61 (0.33, 0.91) | 0.69 (0.42, 0.94) | 0.59 (0.29, 0.89) | 0.81 (0.57, 0.96) | | 0.76 (0.53, 0.94) |
|  |  | p10 | 0.83 (0.62, 0.96) | 0.8 (0.54, 0.96) | 0.8 (0.57, 0.95) | 0.71 (0.45, 0.95) | 0.76 (0.47, 0.94) | 0.69 (0.37, 0.92) | 0.87 (0.7, 0.98) | | 0.83 (0.61, 0.96) |
|  |  | p19 | 0.83 (0.6, 0.95) | 0.82 (0.6, 0.96) | 0.81 (0.56, 0.96) | 0.76 (0.47, 0.94) | 0.78 (0.48, 0.96) | 0.69 (0.42, 0.88) | 0.87 (0.66, 0.97) | | 0.86 (0.64, 0.97) |
|  |  | pSp | 0.86 (0.72, 0.95) | 0.81 (0.59, 0.96) | 0.82 (0.61, 0.95) | 0.72 (0.44, 0.95) | 0.76 (0.51, 0.95) | 0.73 (0.56, 0.92) | 0.93 (0.85, 0.98) | | 0.87 (0.73, 0.95) |
|  |  |  |  |  |  |  |  |  |  | |  |

Table S4: Proportions of variance in model performance explained by the random effect of species and the fixed effects of the number of predictors, the modelling technique and the number of pseudo-absences for spatially independent validation (SIV), and cross-validation (CV).

| **Type of effect** | **Name of effect** | **Mammals** | |  | **Amphibians** | |  | |  | **Fishes** | | |
| --- | --- | --- | --- | --- | --- | --- | --- | --- | --- | --- | --- | --- |
|  |  | SIV | CV |  | SIV | CV | |  | |  | SIV | CV |
| **Random** | Species | 52.55 | 24.59 |  | 55.18 | 29.5 | |  | |  | 64.07 | 32.95 |
|  |  |  |  |  |  |  | |  | |  |  |  |
| **Fixed** | Predictor set | 3.30 | 24.44 |  | 4.85 | 20.46 | |  | |  | 2.89 | 26.31 |
|  | Modelling technique | 1.29 | 10.88 |  | 1.52 | 7.58 | |  | |  | 1.52 | 1.72 |
|  | Niche overlap | 0.00 | 0.00 |  | 0.00 | 0.00 | |  | |  | 6.72 | 5.92 |
|  | Pseudo-absence set | 0.00 | 3.49 |  | 0.02 | 0.19 | |  | |  | 0.34 | 2.54 |
|  | Predictor set * Modelling technique | 0.82 | 14.09 |  | 1.66 | 5.87 | |  | |  | 0.82 | 11.54 |
|  | Modelling technique * Pseudo-absence set | 1.63 | 0.00 |  | 0.23 | 0.80 | |  | |  | 0.00 | 0.00 |
|  | Predictor set * Pseudo-absence set | 0.76 | 0.00 |  | 0.00 | 0.52 | |  | |  | 0.00 | 0.00 |
|  | Modelling techique * Niche overlap | 0.00 | 0.00 |  | 0.44 | 0.21 | |  | |  | 0.19 | 2.11 |
|  |  |  |  |  |  |  | |  | |  |  |  |
| **Residual** | Residual | 39.65 | 22.51 |  | 36.1 | 35.04 | |  | |  | 23.45 | 16.91 |
